# Aggrephagy: Selective Disposal of Protein Aggregates by Macroautophagy

**DOI:** 10.1155/2012/736905

**Published:** 2012-03-22

**Authors:** Trond Lamark, Terje Johansen

**Affiliations:** Molecular Cancer Research Group, Institute of Medical Biology, University of Tromsø, 9037 Tromsø, Norway

## Abstract

Protein aggregation is a continuous process in our cells. Some proteins aggregate in a regulated manner required for different vital functional processes in the cells whereas other protein aggregates result from misfolding caused by various stressors. The decision to form an aggregate is largely made by chaperones and chaperone-assisted proteins. Proteins that are damaged beyond repair are degraded either by the proteasome or by the lysosome via autophagy. The aggregates can be degraded by the proteasome and by chaperone-mediated autophagy only after dissolution into soluble single peptide species. Hence, protein aggregates as such are degraded by macroautophagy. The selective degradation of protein aggregates by macroautophagy is called aggrephagy. Here we review the processes of aggregate formation, recognition, transport, and sequestration into autophagosomes by autophagy receptors and the role of aggrephagy in different protein aggregation diseases.

## 1. Introduction

Misfolded proteins result from mutations, incomplete translation giving defective ribosomal products (DRiPs), misfolding after translation, aberrant protein modifications, oxidative damage, and from failed assembly of protein complexes. Misfolded proteins expose hydrophobic patches that are normally buried internally in the native folded state. These hydrophobic surfaces trigger aggregation and can sequester normal proteins compromising their functionality [1]. To defend cells against the hazards caused by accumulation of misfolded proteins, different protein quality control machineries are active at several levels. Molecular chaperones, like the heat shock proteins (Hsp), recognize, assist folding, prevent aggregation, and attempt to repair misfolded proteins. However, if the damage is beyond repair, chaperone complexes, often in conjunction with interacting ubiquitin E3 ligases, channel the misfolded protein or protein aggregates to degradation pathways.

### 1.1. The UPS

**Figure 1:**
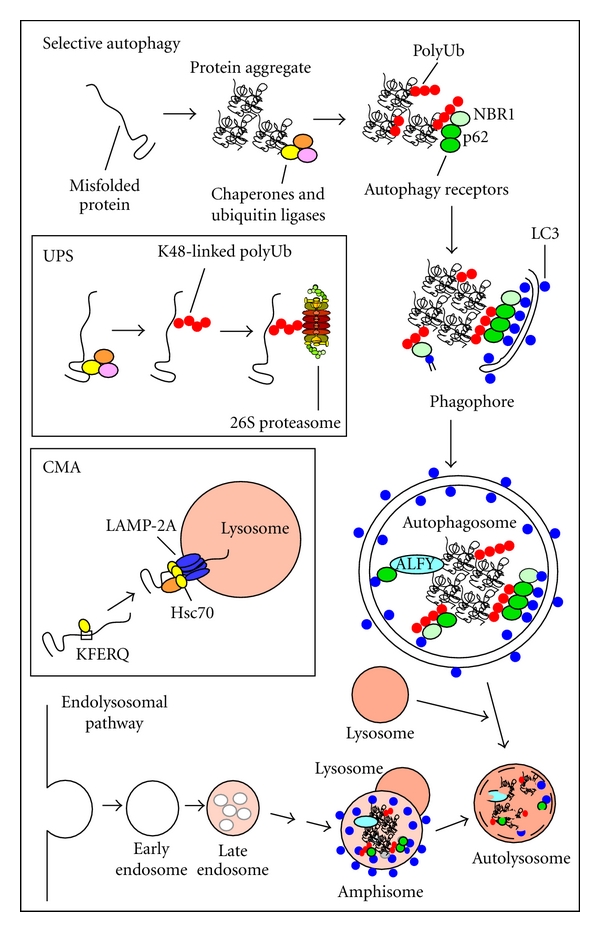
Proteins recognized as misfolded by molecular chaperones can be degraded by selective autophagy, the ubiquitin-proteasome system (UPS) or chaperone-mediated autophagy (CMA). In selective autophagy, misfolded proteins are often assembled into aggregates before they are degraded. They are also often ubiquitinated, and this induces the recruitment of ubiquitin binding cargo receptors such as p62 and NBR1. These cargo receptors bind to ubiquitinated cargos (in this case a protein aggregate) and to ATG8 homologues conjugated to the inner surface of the phagophore (LC3 indicated as blue dots). This way, cargos are selectively delivered to the inner surface of the phagophore. An autophagosome is formed by closure of the phagophore. The autophagosome fuses with a late endosome or with a lysosome, but the end point is in both cases the formation of an autolysosome where the contents are degraded. Substrates for the UPS and CMA degradation pathways need to be in a soluble and monomeric form. Degradation by the UPS depends on K48-linked polyubiquitination of the misfolded substrate. The substrate is then delivered to the 26S proteasome, where it is deubiquitinated and degraded. Degradation by CMA depends on an Hsc70-mediated recognition of a KFERQ motif on the misfolded substrate. The substrate is then delivered to the lysosomal receptor LAMP-2A, transported into the lumen of the lysosome, and degraded.

The two major degradation systems in the cell are the ubiquitin-proteasome system (UPS) and the lysosome (Figure 1). The UPS comprises the proteasome and the enzymatic cascade catalysing the ubiquitination of substrates destined for degradation in the proteasome. The prime tag for proteasomal degradation is a chain of 4 or more ubiquitin moieties covalently linked to lysine residue(s) of the target. Ubiquitin has 7 internal lysines (K6, K11, K27, K29, K33, K48, and K63) that can be linked, forming polyubiquitin chains [2, 3]. K48-linked polyubiquitin chains represent the canonical proteasomal degradation tag, but also K11-linkages are used and some substrates with K63-linked polyubiquitin can be degraded by the proteasome [4]. An enzyme cascade of E1 activation, E2 conjugation, and E3 ligation enzymes mediates the ubiquitination of target proteins [5]. The human repertoire consists of two ubiquitin-specific E1 activation enzymes, about 30 E2 conjugation enzymes, and more than 1000 E3 ligases providing a great versatility in substrate recognition and enabling diversity in ubiquitin chain linkages added to substrates [6–9].

The proteasome consists of a barrel-shaped catalytic core particle, called the 20S proteasome, and the regulatory particle [10, 11]. The cylindrical catalytic particle has a central channel with a diameter of only ~1.5 nm with three proteolytically active proteasomal subunits facing the inside of this channel. Hence, the digestion chamber is inaccessible for folded proteins. Substrate access is regulated by “gates” on both sides of the 20S proteasome. The complete 26S proteasome contains two 19S regulatory subunits, one on each side, mediating substrate recognition, unfolding, and transfer into the catalytic chamber of the 20S proteasome [10–12]. The 19S regulatory particle consists of the base and the lid. The base has six AAA-type ATPases (Rpt1–Rpt6) forming the hexameric ring and four non-ATPase subunits (Rpn1, Rpn2, Rpn10, and Rpn13). The hexameric ring unfolds proteasomal substrates and together with Rpn1-Rpn2 helps open the gate into the catalytic chamber of the 20S proteasome. Rpn10 and Rpn13 recognize and recruit proteasomal substrates by binding to the K48-linked polyubiquitin degradation tag [13]. The lid has nine Rpn subunits (Rpn3, Rpn5–9, Rpn11-12, and Rpn15). Rpn11 is a de-ubiquitination enzyme (DUB) responsible for recycling of ubiquitin [10, 11, 13].

### 1.2. Autophagy

The lysosomal degradation of intracellular contents, such as misfolded proteins, protein aggregates, and organelles, is mediated by autophagy [14, 15]. Three major types of autophagy have been described in mammalian cells, that is, macroautophagy [14–16], microautophagy [17], and chaperone-mediated autophagy (CMA) [18, 19]. Of these, macroautophagy (hereafter referred to as autophagy) is the only process that can mediate the degradation of larger substrates such as organelles, microbes, and protein aggregates (Figure 1). The UPS and CMA are only capable of degrading one extended polypeptide at the time. Autophagy is initiated by the formation of a double-membrane structure, the phagophore. The source of the phagophore membrane is still under debate, and both the ER, mitochondria, plasma membrane, and the Golgi apparatus have been implicated [20]. Elongation of the phagophore depends on two ubiquitin-like conjugation reactions. First, autophagy-related gene 12 (ATG12) is conjugated to ATG5 resulting in the formation of an oligomeric ATG5-ATG12-ATG16L complex. This complex is then needed for the conjugation of ATG8 homologues to phosphatidylethanolamine (PE) on the phagophore membrane [21]. Mammalian ATG8 homologues are grouped into three subfamilies, that is, the LC3 subfamily (LC3A, B, and C), the GABARAP subfamily (GABARAP and GABARAPL1/GEC1), and GABARAPL2/GATE-16 [22]. Conjugation of ATG8 homologues to both sides of the phagophore enables them to act as surface receptors for the specific recruitment of other proteins. Lipidated ATG8 proteins are also involved in membrane biogenesis of autophagosomes via their membrane fusion activity [23]. Autophagosomes are formed by closure of the phagophore into a double-membrane vesicle. Lipidated ATG8 homologues on the outer membrane are released by ATG4B upon completion of autophagosome formation [24]. In mammalian cells autophagosomes often form at the cell periphery and are transported along microtubules and fuse with late endosomes or lysosomes at the microtubule-organizing centre (MTOC) area of the cells finally resulting in degradation of their contents.

### 1.3. Selective Autophagy

Autophagy has been considered as a bulk degradation system with little or no selectivity that is induced to replenish energy stores upon starvation. However, there is now considerable evidence to support the notion that the process may also be highly specific [25–27]. The term selective autophagy refers to the selective degradation of organelles, bacteria, ribosomes, specific proteins, and protein aggregates by autophagy. In selective autophagy, an important role is played by proteins acting as autophagy receptors such as p62 and NBR1 that bind directly to ATG8 homologues (Figure 1). The autophagy receptors are themselves degraded by autophagy, and they mediate selective autophagy via interactions with substrates that are simultaneously degraded [26, 28–31]. Selective autophagy is an important quality control system and is part of a basal constitutive autophagy that can also be induced or boosted by various stressors including oxidative stress, infections, protein aggregation, and proteasomal inhibition [26, 32].

The formation of larger protein aggregates is regarded as a cellular defense mechanism [33, 34]. The large aggregates or inclusions are less toxic to the cell than the presence of smaller microaggregates dispersed throughout the cell [33, 35–38]. Since the large inclusions are usually readily visible in the light microscope, while the more toxic soluble species are not, the inclusions can also be used to distinguish between different neurodegenerative disorders involving aggregation of specific, often mutant, proteins. The protein aggregates may also represent intermediates in autophagic degradation of aggregation-prone proteins [39]. The assembly of autophagy substrates into larger aggregates or clustered structures is a common feature of selective autophagy [26]. It may facilitate their uptake into autophagosomes, and aggregates may work as nucleation sites for the phagophore, the forming isolation membrane [40].

Proteins damaged beyond repair are recognized and sorted by chaperone and co-chaperone complexes containing chaperone-assisted ubiquitin E3 ligases to three different degradation pathways: the UPS, CMA, and/or aggrephagy. The term aggrephagy was introduced by Per Seglen to describe the selective sequestration of protein aggregates by autophagy [41]. In the following we will review the current knowledge on how protein aggregates are recognized, sorted, and degraded by aggrephagy.

## 2. Crosstalk between Degradation Pathways: Hsp70/Hsp90 and Co-Chaperones

### 2.1. Quality Control of Newly Synthesized Proteins

**Figure 2:**
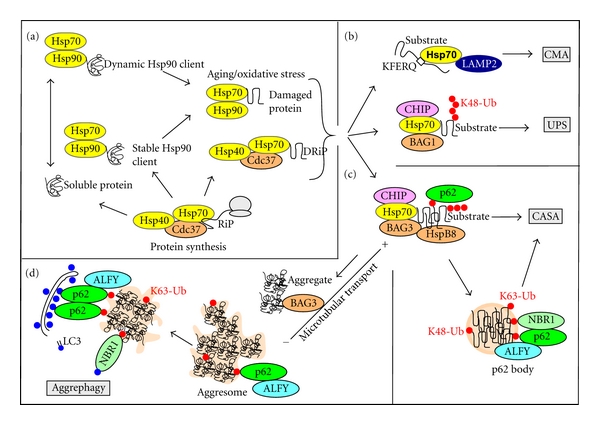
Protein degradation assisted by heat shock proteins and their co-chaperones. (a) Substrates selected for degradation by heat shock proteins are either defective ribosomal products (DRiPs) or Hsp90 client proteins that start to unfold or aggregate. Formation of the latter type of substrate is increased under conditions of oxidative stress or during aging. (b) Misfolded and monomeric substrates bound to Hsp70/Hsc70 are preferentially degraded by CMA or by the UPS. (c) In response to aggregation, or if the capacity of CMA and the UPS is insufficient, substrates are degraded by chaperone-assisted selective autophagy (CASA). This process relies on the co-chaperones BAG3 and HspB8, the E3 ubiquitin ligase CHIP, and autophagy receptors such as p62. The process may also rely on the assembly of the misfolded substrates into p62 bodies. (d) If degradation of misfolded substrates is impaired, BAG3 interacts with dynein and transport protein aggregates along microtubules to the aggresome. The contents of aggresomes may subsequently be degraded by aggrephagy.

**Figure 3:**
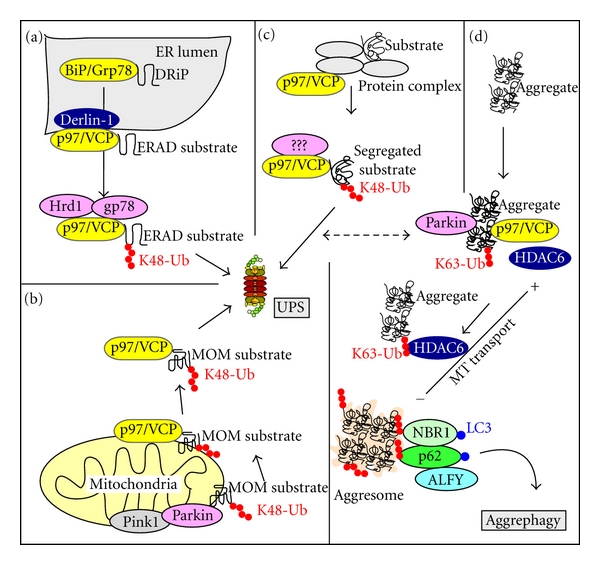
Protein degradation assisted by p97/VCP and HDAC6. (a) Misfolded substrates located in the ER lumen or at the ER membrane are recognized by the ER luminal Hsp70 homologue BiP/Grp78 and degraded by ER-associated degradation (ERAD). A complex of p97/VCP and Derlin-1 mediates the transport of ERAD substrates into the cytoplasm where they are ubiquitinated by E3 ligases such as Hrd1 and gp78 and degraded by the UPS. (b) p97/VCP mediates the segregation of ubiquitinated mitochondrial outer membrane (MOM) substrates into the cytoplasm, where they are degraded by the UPS. (c) p97/VCP mediates the segregation of selected substrates from nuclear or cytoplasmic protein complexes, followed by their degradation by the UPS. (d) p97/VCP is also required for the transport of protein aggregates to the aggresome. This depends on ubiquitination of the aggregate by an E3 ligase such as Parkin, and the delivery of the ubiquitinated aggregate to HDAC6. HDAC6 binds to K63-linked polyubiquitin chains and to dynein, and it is responsible for the transport of ubiquitinated protein aggregates along microtubules to the aggresome. The contents of aggresomes may subsequently be degraded by aggrephagy.

A complex consisting of Hsp70, Hsp40, and several co-chaperones including Cdc37 mediates the protein quality control of newly synthesized proteins in the cytosol (Figure 2(a)). In this process, DRiPs and aggregation-prone translational products are degraded. Functional products are released or delivered to the Hsp90 chaperone complex. In ER and mitochondria, homologs of Hsp70 play a similar role in the quality control of newly synthesized proteins. The protein quality control in ER (reviewed in [42]) begins when a nascent chain enters ER through the translocon. Newly synthesized proteins transiently undergo cycling with the ER luminal Hsp70 paralog BiP/GRP78 which is associated with several co-chaperones. Proteins that are recognized as misfolded or not properly processed are delivered for ER-associated degradation (ERAD). ERAD substrates are retranslocated into the cytoplasm where they are degraded mainly by the UPS (Figure 3(a)). A chaperone holdase activity mediated by an associated BAG6 complex is needed to keep ERAD substrates unfolded, yet soluble, until they are degraded [43].

### 2.2. Selective Degradation of Damaged Proteins

Quality control of mature proteins is another important role of Hsp70/Hsp90 chaperone complexes (Figure 2(a)). There is considerable crosstalk between the Hsp70 and Hsp90 chaperone complexes, but in general Hsp90 protects proteins from unfolding and aggregation, whereas Hsp70 is responsible for their degradation in cases when unfolding or aggregation cannot be prevented. The classic clients of Hsp90 are unstable proteins that undergo tight cycling with the chaperone, and in response to Hsp90 inhibition, these proteins are rapidly delivered to Hsp70 and degraded. Other more stable proteins may be less dependent on Hsp90, but they may still undergo dynamic cycling with the chaperone complex [44].

If a misfolded protein cannot be refolded by chaperones, this normally results in its degradation by the UPS, CMA, and/or selective autophagy. Since Hsp70 can mediate the delivery to all three degradation pathways, the same substrate can in principle be degraded by all three systems (Figures 2(b) and 2(c)). Inefficient degradation by one system is often compensated by increased degradation by another system. Impairment of the UPS or CMA leads to activation of autophagy [45–49]. Vice versa, in cells where autophagy is inhibited, CMA is increased to compensate [50].

Previously, autophagy was considered to act only as a back-up system when the capacity of UPS and CMA is overwhelmed. However, selective autophagy is active also under normal conditions, and tissues such as brain, liver, and muscle have a constitutive need for selective autophagy [51–55]. An obvious role for selective autophagy under normal conditions is to degrade substrates that are not solubilized or unfolded and exist as some form of aggregated structure.

### 2.3. Degradation by CMA or the UPS

In CMA, cytosolic substrates with a KFERQ-like motif are degraded in lysosomes without the formation of autophagic vesicles (Figure 1). Substrates are recognized by an Hsc70 complex, delivered to the lysosomal receptor LAMP-2A, and transported into the lumen of the lysosome where they are degraded [18, 19]. The KFERQ-like motif is present in 30% of cytosolic proteins, and the fraction may be higher than this due to posttranslational modification [56]. CMA activity is proportional to the level of LAMP-2A at the lysosomal membrane. Expression of LAMP-2A is upregulated, and CMA therefore increased under oxidative stress conditions [57].

In order to be degraded by the UPS, a substrate must be polyubiquitinated with chains consisting of four or more preferably K48-linked ubiquitin moieties. CHIP (carboxyl terminus of constitutive Hsc70-interacting protein) is a cofactor for Hsp70 and Hsp90 and a prototype of the chaperone-dependent ubiquitin E3 ligases involved in proteasomal degradation of Hsp90 client proteins [58–60]. The DUB ataxin-3 regulates the length of ubiquitin chains added to CHIP substrates, and it is likely that this ubiquitination is not only regulated by CHIP but also by other chaperone-assisted E3 ligases and DUBs [61].

### 2.4. Chaperone-Assisted Selective Autophagy (CASA)

The group of Hohfeld has introduced the term chaperone-assisted selective autophagy (CASA) to describe selective autophagy of misfolded proteins following a chaperone-mediated formation of protein aggregates that are targeted to form autophagosomes [62]. The dedicated chaperone in CASA is BAG3 (Figure 2(c)). The BAG (Bcl2-associated athanogene) family (BAG1-6) of co-chaperones uses their BAG domain to interact with the ATPase domain of Hsp70. BAG1 competes with Hip for interaction with Hsp70, and binding of BAG1 induces proteasomal degradation of misfolded Hsp70 substrates (Figure 2(b)). Alternatively, a multi-chaperone complex of Hsp70, BAG3, and HspB8 induces selective degradation of misfolded proteins by autophagy. Substrates shown to be degraded by this complex include polyQ-expanded huntingtin [63] and SOD1 [64]. CASA is important also under normal growth conditions, and mice deficient for BAG3 die shortly after birth due to the development of a progressive muscle weakness [65]. In muscles, a complex containing BAG3, its partner HspB8, CHIP, and Hsp70 is constitutively needed for the maintenance of Z-disks [66]. Loss of BAG3 activity in patients or transgenic animals leads to a contraction-dependent disintegration of Z-discs [65, 67]. The BAG3 complex is here needed for clearance of damaged components such as filamin [66].

### 2.5. p62 Bodies, DALIS, and ALIS

There is an intimate relationship between CASA and the formation of p62 bodies (Figure 2(c)), but more studies are needed to verify whether their formation is required for CASA or not. The contents of p62 bodies are degraded by selective autophagy, and this depends on a direct interaction of its major constituent p62 and its interaction partner NBR1, with ATG8 homologues on the phagophore [30, 31]. The decision to form p62 bodies and to degrade misfolded substrates by CASA may be decided by the BAG3 : BAG1 ratio within the specific cell. The link between BAG3 and the formation of p62 bodies was initially described by the group of Christian Behl [68]. Strikingly, in aging cells, an increased level of BAG3 relative to BAG1 is responsible for a shift from proteasomal towards autophagic degradation of misfolded proteins. This correlates with an increased formation of p62 bodies [68].

A specialized type of protein aggregate clearly related to p62 bodies is the dendritic cell aggresome-like induced structures (DALISs) initially studied by the group of Philippe Pierre [69, 70]. This type of ubiquitinated structure is transiently formed in antigen-presenting cells such as dendritic cells and macrophages during immune cell maturation. By using puromycin to induce the formation of DRiPs, they showed that misfolded proteins accumulate in DALIS and become ubiquitinated within these structures. DALIS is an ordered type of structure distinct from aggresomes. The formation of DALIS is stress-induced and transient and does not depend on transport along microtubules [69, 70]. Later studies showed that similar structures can be formed in many cell types in response to stressors like puromycin, oxidative stress, starvation, and transfection, and they were therefore given the name ALIS [71]. We noted that p62 is a major protein in these structures and realized that ALIS and p62 bodies are indistinguishable structures [31]. The relationship between p62 bodies and DALIS needs to be analyzed more carefully. p62 bodies have been used by us as a term to describe aggregates formed by p62 in response to various stressors. A major role of p62 bodies is to serve as substrates for selective autophagy. It is important to realize that some types of p62 bodies may not be true ALIS, in the sense that they may not fulfill the criteria as has been described for the DALIS of dendritic cells [69, 70, 72]. We therefore consider p62 bodies to represent a more broad type of structure, also including aggregates that are different from DALIS/ALIS.

An important role of DALIS during immune cell maturation is MHC class I presentation, and this depends on proteasomal degradation [73]. This actually also occurs for DRiPs accumulated in ALIS in HeLa cells during autophagy inhibition, although these DRiPs are normally also autophagy substrates [74]. A recent study explored the degradation of DALIS formed in dendritic cells, and this study revealed that the contents of DALIS can be degraded both by the proteasome and by selective autophagy [72]. In line with other studies, BAG3 is needed for selective autophagy of these structures, but not for their proteasomal degradation [72]. Degradation by the UPS is probably not a specific feature of the DALIS but may occur with p62 bodies of other cell types as well [71]. However, oxidative stress-induced p62 bodies in pancreatic cells of diabetic rats are only cleared by autophagy [75]. Possibly, the aggregation status of proteins within the p62 body is an important parameter that may regulate the recruitment of BAG3 to the aggregate.

## 3. The Decision to Form Aggregates

### 3.1. The Aggresome

The aggresome, formed in response to proteasomal inhibition or overexpression of aggregation prone proteins, is currently the best-studied protein aggregate with respect to formation and degradation mechanisms. The aggresome is located close to the nuclear envelope at the microtubule organizing center (MTOC), and its formation depends on microtubule-dependent transport of protein aggregates [34, 76]. It is insoluble and metabolically stable. The proteins of an aggresome are normally ubiquitinated [77], and they are enclosed by intermediate filaments such as vimentin and keratin, depending on cell type [34, 76]. Other types of inclusions observed in proteinopathies may have a nuclear or more dispersed cytoplasmic location. The formation of a specific type of inclusion is often associated with the formation of a variety of smaller intermediates that can be unstructured or have a variety of different types of structures [1]. A study reported two different types of aggresome-like structures found both in yeast and mammalian cells called the “juxtanuclear quality control” (JUNQ) and “insoluble protein deposit” (IPOD) that differ from the classical definition of an aggresome since they do not localize to the MTOC [78]. Both of these structures require microtubular transport for their formation. IPODs are localized to the cell periphery often near vacuoles, and the aggregates do not contain ubiquitin. JUNQ, on the other hand, is localized close to the nucleus and contains ubiquitinated proteins and associated proteasomes. More studies are required to determine the relationships between these different “aggresomes.”

What should be noted is that aggregation may also be part of an important functional state of some proteins. One example is the autophagy receptor p62 which is present in almost all types of protein aggregates. p62 is continuously degraded by autophagy, and this relies on its ability to polymerize [29]. What type of overall structure p62 forms in order to be degraded is not known, but this is a relevant question since this intrinsic structure may also be essential for the structural and functional role of p62 in protein inclusions. Proteins like p62, ALFY (autophagy-linked FYVE protein), and likely also NBR1 (neighbor of BRCA1 gene), are general contents of protein inclusions and are believed to be there because they are involved in both their construction and their degradation by autophagy [26, 28, 30, 31, 79–81]. There is also an ongoing discussion whether it is the mature inclusions or the intermediate precursors that are degraded by autophagy. Two independent pathways have so far been described for the formation of an aggresome, distinguished by whether it is histone deacetylase 6 (HDAC6) or BAG3 that mediates the actual transport of aggregates to the aggresome (Figures 2(d) and 3(d)).

### 3.2. HDAC6: Transport of Aggregates and Autophagosomal Maturation

HDAC6 facilitates dynein-mediated transport of ubiquitinated substrates to the aggresome, and it is also important for the clearance of aggresomes by autophagy [77, 82, 83] (Figure 3(d)). These roles of HDAC6 are in particular important under conditions when proteasomal degradation is impaired and misfolded proteins are preferentially degraded by autophagy [46, 84]. HDAC6 interacts directly with dynein and with ubiquitinated substrates and has a preference for K63-linked polyubiquitin chains [83, 85]. In addition to a role in aggregate formation and aggrephagy, HDAC6 has a role in maturation of autophagosomes and knockdown of HDAC6 results in the accumulation of autophagosomes [86]. These autophagosomes contain ubiquitinated proteins, demonstrating a role for HDAC6 in the maturation of a subset of autophagosomes involved in selective autophagy of misfolded proteins. The role of HDAC6 in this process is to regulate the actin cytoskeleton [86]. HDAC6 and p62 may act sequentially in the degradation of ubiquitinated protein aggregates with p62 recruiting phagophores to the aggregates for autophagosome formation and HDAC6 acting at the maturation step enhancing fusion between autophagosomes and lysosomes by remodeling actin [83].

### 3.3. BAG3-Mediated Formation of Aggresomes

BAG3 and CHIP are both needed for targeting of Hsp70 substrates to the aggresome [38] (Figure 2(d)). BAG3 interacts directly with dynein, and this directs transport of Hsp70 substrates to the aggresome [87]. This transport does not depend on ubiquitination of substrates, although the E3 ubiquitin ligase CHIP is required [38]. Depletion of CHIP inhibits aggresome formation in response to proteasomal inhibition, whereas expression of a dominant negative CHIP that does not interact with E2 conjugation enzymes induces aggresome formation [38]. Hence, in the absence of proteasomal degradation or CHIP-mediated ubiquitination, CHIP induces aggregation and BAG3-mediated transport of misfolded substrates, resulting in the formation of aggresomes. Hence, BAG3 may play an important role in recruiting non-ubiquitinated substrates to the aggresome [87].

### 3.4. p97/VCP: An Ubiquitin-Associated Hsp-Independent Molecular Chaperone

The AAA-ATPase family protein p97/VCP (valosin-containing protein) is a molecular chaperone with important roles in cell division, organelle biogenesis, nuclear envelope formation, and protein degradation [88]. To understand the diversity of cellular roles displayed by p97/VCP, it is important to look at the roles of the various cofactors it interacts with. Most of these interactions are mediated by the N-terminal domain, while a few are mediated by the C-terminal 10 amino acids [89]. Functional roles are known only for a subset of these interactions, but the majority of p97/VCP cofactors have a clear connection to ubiquitin. Loss of p97/VCP in mammalian cells results in accumulation of insoluble ubiquitinated proteins [90–92]. Functional p97/VCP is a homohexamer, and ATP hydrolysis is associated with conformational changes and release of substrates and cofactors [93]. Several studies support a “segregase” activity of p97/VCP, in which ATP hydrolysis is used to segregate ubiquitinated substrates from protein complexes, cell membranes, and chromatin [94–96] (Figures 3(a)–3(c)). p97/VCP is located in the cytoplasm and nucleus and is recruited to the ER membrane in response to ER stress. In ERAD, p97/VCP interacts with the integral ER membrane protein Derlin-1 to unfold, transfer, and extract UPS substrates from the ER membrane [97] (Figure 3(a)). Several ERAD-directed E3 ligases have been detected in mammalian cells, including Hrd1 and gp78 [98]. p97/VCP also plays a role in autophagic degradation of damaged mitochondria after treatment with CCCP (Figure 3(b)). In this case, it is needed for the extraction and delivery of mitochondrial mitofusins to the UPS, after they first have been ubiquitinated by Parkin [99, 100].

No crosstalk between p97/VCP and the Hsp70/Hsp90 molecular chaperones is reported, and cellular roles mediated by p97/VCP may therefore be distinct from those displayed by the other group of molecular chaperones. Interestingly, overexpression of p97/VCP inhibited accumulation of ubiquitinated proteins in autophagy deficient cells overexpressing p62, indicating that there may be a competitive relation between p62 and p97/VCP for the binding to ubiquitinated substrates [101]. p97/VCP is important for aggresome formation in mammalian cells in response to proteasomal inhibition [92, 102–104]. p97/VCP is proposed to induce aggresome formation via a delivery of ubiquitinated protein aggregates to HDAC6 (Figure 3(d)), but the relation between p97/VCP and HDAC6 in this process is only partially understood [105]. Similar to HDAC6, p97/VCP is involved in maturation of autophagosomes. Knockdown of p97/VCP leads to accumulation of autophagosomes containing ubiquitinated substrates [106].

Mutations in p97/VCP cause inclusion body myopathy associated with Paget's disease of the bone and frontotemporal dementia (IBMPFD) [107]. Structural data for p97/VCP mutants reveals conformational changes in the N-terminal domain [108]. This has a strong effect on cofactor interactions, and some interactions like binding of the ubiquitin ligase E4B are reduced whereas others, like binding of the DUB ataxin 3, are increased [109]. Myoblasts expressing IBMPFD mutants of p97/VCP have defects in degradation of ERAD substrates [110], and their expression in myoblasts or transgenic mouse muscle leads to accumulation of ubiquitinated aggregates [110, 111]. Aggresome formation in cell culture is also affected by mutant p97/VCP expression [102, 103]. FRAP analyses suggest that it is the release of substrates from p97/VCP that is impaired, so that aggregation-prone proteins are not delivered to HDAC6 and therefore accumulate in peripheral aggregates lacking p62 and LC3 [102]. The failure to form aggresomes could be rescued by HDAC6 overexpression [102], suggesting that HDAC6 has a protective role.

### 3.5. Ubiquilin-1: A Ubiquitous Distributor and Chaperone

Ubiquilin-1 is another protein linked to the sorting of misfolded proteins to different degradation systems. But ubiquilin-1 is also a chaperone needed for folding and stabilization of specific client proteins. The four mammalian ubiquilins have a domain structure reflecting that of p62, with a ubiquitin binding C-terminal UBA domain and an N-terminal UbL domain interacting with the Rpn10/S5A proteasomal subunit [112, 113]. Ubiquilin-1 is involved in ERAD as part of a complex with erasin and p97/VCP [114]. More recent studies have indicated a role for ubiquilin-1 in delivery of proteins to CMA or to autophagy. Ubiquilin-1 is itself degraded by both pathways [115]. Ubiquilin-1 is also involved in the delivery of proteins to the aggresome [116–118], and it protects against polyQ-induced cell death in cellular and invertebrate models of Huntington's disease [119, 120]. It may also promote autophagic degradation of protein aggregates [121, 122]. Ubiquilin-1 has an intrinsic chaperone activity in vitro [123], and it seems to act as a chaperone for the aggregation-prone amyloid precursor protein (APP) [123]. In HeLa cells, expression of ubiquilin-1 reduces toxicity associated with APP and protects against aggregation of APP [123]. Brains of Alzheimer's disease (AD) patients often have a decreased level of ubiquilin-1 which may contribute to late-onset AD [123].

## 4. Linking Protein Aggregates with the Phagophore

### 4.1. p62 and NBR1

Selective autophagy depends on autophagy receptors like p62 and NBR1. These proteins are themselves degraded by autophagy due to a direct interaction with ATG8 family proteins conjugated to PE and bound to the phagophore membrane [26, 28–31]. p62 and NBR1 share a similar domain architecture, both containing an N-terminal PB1 domain and a C-terminal UBA domain [124]. The interaction between p62 or NBR1 and ATG8 homologues is mediated via a short, linear LIR motif in p62 and NBR1 [29–31, 125, 126]. The p62 LIR has the core motif DDDWTHL. Following the initial discovery of an LIR in p62 [31], this motif has been identified in an increasing list of proteins. Based on characterized LIR motifs, the present consensus sequence is D/E-D-W/F/Y-x-x-L/I/V [26]. The LIR interaction surface of ATG8 proteins has two hydrophobic pockets accommodating the aromatic (W/F/Y) and the hydrophobic side chains (L/I/V) of the core motif, and the acidic residues often interact with basic residues of the N-terminal arm of the ATG8s [29, 125–127]. Lipidated ATG8 proteins are located both on the inner and outer surfaces of autophagic vesicles, and they therefore make a perfect scaffold for specific recruitment of proteins to the phagophore or the autophagosome. p62 is a polymeric protein, and polymers are made via head-to-tail interactions between PB1 domains [124, 128]. The PB1 domain-mediated polymerization is essential for the selective degradation of p62 by autophagy [29], and it is required for the targeting of p62 to the autophagosome formation site at the ER [40]. It is also crucial for the ability of p62 to assemble proteins into aggregates [28]. p62 and NBR1 have a very different primary sequence, and NBR1 contains several domains that are not present in p62. Homologs of NBR1 are found throughout the eukaryotic kingdom, whereas the presence of p62 is unique for metazoans and likely the result of a duplication event early in the metazoan lineage [129]. Plant NBR1 is able to polymerize via the PB1 domain, and this is required for autophagic degradation similar to metazoan p62 [129]. During evolution, NBR1 has lost the ability to polymerize via the PB1 domain, while p62 has lost several domains like the FW domain. Therefore these two proteins may have independent roles in selective autophagy, but they may also cooperate as indicated by the fact that p62 interacts directly with NBR1 [124]. Recently, several other autophagy receptors in addition to p62 and NBR1 have been identified. These are ATG32 and NIX/BNIP3L acting in mitophagy [130–132], NDP52 and optineurin in xenophagy [133, 134], and Stbd1 in degradation of glycogen [135]. However, only p62 and NBR1 have so far been linked to degradation of protein aggregates.

It should be noted that p62 is also involved in selective autophagy of non-ubiquitinated substrates, and selective autophagy of a mutant superoxide dismutase 1 (SOD1) causing ALS depends on a direct and ubiquitin-independent interaction between SOD1 and p62 [136]. Recently, p62 was found to be required for selective autophagic clearance of a non-ubiquitylated substrate, an aggregation-prone isoform of STAT5A (STAT5A_ΔE18) that formed aggresomes and/or aggregates by impairment of proteasome functioning or autophagy [137]. Different domains of p62 interacted with SOD1 and STAT5A in these cases. A third example of p62-mediated selective autophagy of non-ubiquitin substrates is the p62-mediated autophagic clearance of Sindbis virus capsids from neurons of infected mice [138]. A bona fide example of ubiquitin-independent aggrephagy is seen in *C. elegans* where the polymeric autophagy receptor SEPA-1 binds to the P granule component PGL-3 and to the ATG8 homologue LGG-1 to mediate the selective autophagic degradation of P granules [139]. This way the maternally derived germ P granule components are degraded by aggrephagy in somatic cells during embryogenesis. However, in most cases ubiquitin binding seems to be important and a study on the role of p62 and ubiquitin in pexophagy clearly indicates that ubiquitin may serve as a label that is recognized by p62 [140]. Less is known about the role of NBR1 in selective autophagy, mainly because it is less studied. p62 has been implicated in the autophagic clearance of midbody ring complexes [141], and recently NBR1 was found to be required and more important than p62 for clearance of midbody derivatives by autophagy [142]. It was found that disposal of midbody derivatives accompanied stem-cell differentiation and that the autophagy receptor NBR1 bound to the midbody protein CEP55 to mediate the autophagic degradation.

### 4.2. ALFY

ALFY is a 400 kDa scaffold protein with an ensemble of domains located to its C-terminal region. This part of ALFY contains a BEACH domain, an ATG5-interacting WD40 repeat region [80], and a PtdIns(3)P-binding FYVE domain [143]. Co-immunoprecipitation experiments indicate that the BEACH domain in ALFY is important for its ability to form complexes in vivo with p62 [79]. ALFY and p62 colocalize strongly in cytoplasmic and nuclear protein aggregates in cell culture [79, 80], and they are both degraded by autophagy in response to the formation of p62 bodies in HeLa cells [79]. In fact, p62, NBR1, and ALFY are all important for selective autophagy of p62 bodies in HeLa cells [30, 31, 79].

ALFY is under normal conditions mainly nuclear. In response to amino acid starvation or puromycin-induced accumulation of DRiPs, ALFY is redistributed to cytoplasmic p62 bodies [79] (Figure 2(d)). It is also redistributed to cytoplasmic polyglutamine inclusions [80] and to aggregates induced in response to proteasomal inhibition [143]. The redistribution of ALFY in HeLa cells depends on p62 and seems to depend on the ability of p62 to shuttle between the cytoplasm and the nucleus [79]. Autophagic degradation of ALFY most likely depends on its association with p62 and/or cytoplasmic protein aggregates [79, 80].

ALFY is required for aggrephagy, but not for starvation-induced autophagy [80]. Knock-out studies have revealed an important role of ALFY in constitutive autophagy of misfolded proteins, both in mammals and in flies [80, 144]. In flies, knockout of the *Drosophila* homologue *blue cheese* (bchs) results in accumulation of ubiquitinated protein inclusions, neurodegeneration, and a reduced life-span [144]. Furthermore, overexpression of Bchs reduces neurotoxicity in a *Drosophila* eye model of polyglutamine toxicity [80]. In mammals, ALFY is recruited to cytoplasmic and nuclear protein inclusions as part of a complex containing p62, NBR1, LC3, ATG5, ATG12, and ATG16L [80]. In mammalian cell culture, ALFY is required for efficient degradation of polyglutamine and *α*-synuclein inclusions. This depends on a direct interaction between ALFY and ATG5 [80]. Notably, overexpression of the C-terminal part of ALFY alone promoted degradation of polyglutamine inclusions in a neuronal lentiviral model [80]. Very likely, co-recruitment of p62, NBR1, and ALFY and their interaction partners, to a protein aggregate, initiates the formation of autophagy membranes. However, more studies are needed to identify the specific roles mediated by each of these proteins recruiting different components of the autophagy machinery for autophagosome formation at the protein aggregate.

## 5. Regulation of Aggrephagy by Posttranslational Modifications

The selective autophagy of protein aggregates is regulated at the level of the autophagic machinery, the level of autophagy receptors, like p62, NBR1, and ALFY, and the level of the protein aggregates [81, 145]. Hitherto, there is a scarcity of data available to illuminate the mechanisms involved in regulating aggrephagy. However, both autophagy receptors and substrates are regulated by posttranslational modifications including ubiquitination, phosphorylation, and acetylation [146].

K63-linked polyubiquitin chains have been associated with autophagic degradation [147, 148], and this may clearly have a role in recruiting autophagy receptors like p62 and NBR1 [30, 149] or HDAC6 [85]. So, is there a simple ubiquitin-code where substrates tagged with K48-linked ubiquitin chains are degraded by the UPS while aggregated substrates tagged with K63-linked ubiquitin are degraded by autophagy? Several of the proteins involved in aggrephagy, like p62 and HDAC6, bind preferentially to K63-linked ubiquitin [85, 149]. The E3 ligase TRAF6 interacts with p62, and it also catalyzes K63-linked ubiquitination of its substrates [150]. Formation of aggresomes requires the activity of the deubiquitinating enzyme ataxin-3 which can bind to HDAC6 and trims both K48- and K63-linked ubiquitin chains [151, 152]. Hence, ataxin-3 (and other DUBs) may be required for editing the ubiquitin code to one favouring aggrephagy [83]. Note that mutant polyQ-expanded ataxin-3 is an aggregate-prone protein that causes spinocerebellar ataxia type 3 that is degraded by autophagy in a mouse model of this disease [153]. The DUB cylindromatosis tumor suppressor (CYLD) interacts with TRAF6 to remove K63-linked ubiquitin in a p62-dependent manner [154]. Hence, in addition to binding to ubiquitinated aggregates, p62 may also be involved in regulating the K63-linked ubiquitination of aggregates acting as autophagy substrates through its interactions with TRAF6 and CYLD. In brains of p62 KO mice, there was a hyperaccumulation of K63-linked ubiquitin in the insoluble fraction suggesting accumulation of substrates and also dysregulation of the TRAF6-CYLD interplay in the absence of p62 [154]. These mice showed AD-like symptoms, and aggregated K63-ubiquitinated tau protein was recovered from brain fractionation experiments [155]. Not surprisingly, TRAF6 is found in the Lewy bodies in sporadic Parkinson's disease (PD) brains [156]. TRAF6 also regulates autophagy positively by mediating K63 ubiquitination of beclin 1, and this is opposed by the DUB A20 [157].

The autophagy pathway is directly regulated by several kinases including Ulk1/2, mTOR, AMPK, and PKA. In addition, autophagy receptors like p62 and optineurin, as well as LC3B, have recently been shown to be regulated by phosphorylation [134, 158, 159]. PKA-mediated phosphorylation of a site in the N-terminal arm of LC3 inhibited its recruitment to autophagosomes [158]. TANK binding kinase 1 (TBK1) phosphorylated optineurin on Ser-177 in the LIR motif, enhancing LC3 binding affinity and autophagic clearance of cytosolic *Salmonella* showing that the LIR-LC3 interaction can be regulated by phosphorylation [134]. Phosphorylation of p62 on Ser-403 in the UBA domain increased the affinity for polyubiquitin and stimulated aggrephagy of ubiquitinated proteins [159]. Interestingly, recently a number of reports show that, similar to p62, also optineurin is found in ubiquitin-positive inclusions in sporadic and familial ALS, neurofibrillary tangles and dystrophic neuritis in AD, and LBs in PD and more neurodegenerative diseases (see that is, refs. [160–162]). Optineurin was recently found to be mutated and causatively linked to the disease in some cases of familial ALS [160]. Mutations of optineurin have also been found in sporadic ALS [163].

The aggregating substrates can be phosphorylated in a manner affecting their clearance. A number of studies report on phosphorylations affecting cleavage, aggregation, and clearance of aggregation-prone polyQ-expanded proteins including huntingtin and ataxin-1 and 3 (see [81]). It has long been recognized that phosphorylation of tau affects its aggregation. In the brains of adult p62 knock-out mice, an age-dependent increase in the activity of several kinases, including glycogen synthase kinase 3*β* (GSK3*β*), protein kinase B (PKB), mitogen-activated protein kinases, and c-Jun-N-terminal kinase, results in hyperphosphorylated tau and formation of neurofibrillary tangles [155].

Members of the basic autophagy apparatus including Atg5, 7, 8, and 12 can be acetylated by the acetyltransferase p300, and p300 binds directly to Atg7 [164]. Acetylation of these proteins mediated by p300 inhibits autophagy, and silencing of p300 increases autophagy flux [164]. Acetylation was also recently shown to affect the autophagic clearance of a fragment of mutant huntingtin and an N-terminal caspase-7 cleavage fragment of ataxin-7. Acetylation of lysine 444 (K444) increased autophagic degradation of mutant huntingtin [165]. This acetylation also mitigated the toxic effects of mutant huntingtin in primary striatal and cortical neurons and in a transgenic *C. elegans* model of Huntington's disease. Mutant huntingtin resistant to acetylation accumulated and led to neurodegeneration in cultured neurons and in mouse brain [165]. The opposite effect of acetylation was seen for ataxin-7 [166]. Cleavage of ataxin-7 by caspase-7 generates toxic N-terminal polyQ-containing fragments that accumulate with disease progression. Acetylation of lysine 257 (K257) adjacent to the caspase-7 cleavage site of ataxin-7 promotes accumulation of the fragment, while the unmodified ataxin-7 fragments are degraded by autophagy [166].

## 6. Other Regulatory Aspects

So far very little is known about differential gene regulation occurring as a result of aggregate formation. There is clearly a link between aggregate formation and oxidative stress responses. p62 binds to the cytoplasmic inhibitor KEAP1 to stabilize the oxidative stress response transcription factor Nrf2, which then induces a repertoire of oxidative stress response genes [167–170]. The p62 gene is itself one of the targets of Nrf2 enabling p62 to set up a positive feedback loop [168]. Deprenyl, which is a candidate neuroprotective drug in PD, can also lead to nuclear accumulation of Nrf2 and induction of oxidative stress response genes [171]. Transcription of the p62 gene is increased during aggregate formation induced by proteasomal inhibition [172]. Activation of the p62-Nrf2 pathway may therefore be an important protective response during aggregate formation and a target for development of neuroprotective drugs.

Several central proteins involved in aggrephagy including Beclin 1, diabetes- and obesity-regulated gene (DOR), p62, and ALFY shuttle between the nucleus and cytosol (see [81]). The nucleocytoplasmic shuttling of p62 is regulated by phosphorylation sites at or C-terminal to the major nuclear localization signal [173]. In the nucleus both p62 and ALFY may be involved in collecting ubiquitinated proteins in the PML (promyelocytic leukemia) nuclear bodies for proteasomal degradation [173]. Whether substrates can be transported out of the nucleus for degradation by autophagy is an open question.

## 7. Is the Aggregate Eaten in One Big Bite or in Smaller Pieces?

It is now well established that autophagy is needed for the removal of cytoplasmic protein inclusions [80, 82, 174–176]. But are the insoluble inclusions solubilized or modified before engulfment? There seems to be a putative conflict between the caging of protein aggregates within intermediate filaments such as vimentin or keratin [34, 76] and the degradation of these aggregates by autophagy. However, knock-out studies of genes associated with aggregation indicate a positive role for aggregation in autophagy [82]. In AD, autophagosomes with electron dense amorphous or multilamellar contents accumulate in massive numbers [177]. The accumulation of protein aggregates or inclusions has also been demonstrated in cell culture. Immunoelectron microscopy on mammalian cells stably expressing Htt103Q revealed that insoluble polyglutamine inclusions are found within autophagosomes [80]. SDS-insoluble Htt103Q was in this case also found in autophagosome fractions after cell fractionation, clearly demonstrating that insoluble inclusions are indeed engulfed by autophagic structures. Also p62 bodies have been shown by immunoelectron microscopy to accumulate inside autophagosomes [31]. It is in this context relevant that other large cellular structures such as organelles and bacteria are degraded by selective autophagy [26], and there is no evidence that these structures are cut into smaller pieces before they are engulfed.

In non-metazoan species, including plants and fungi, Hsp104 forms a complex with Hsp70 and Hsp40 that has disaggregation activity capable of dissolving amyloid-like structures [178]. No homolog of Hsp104 exists in metazoans, but a less potent disaggregation activity was recently shown for a mammalian complex consisting of Hsp110, Hsp70, and Hsp40 [179]. The observed presence of insoluble inclusions within autophagic vesicles does not exclude that another route of degradation is more important, and studies on degradation of aggregation-prone proteins are often confused by the fact that their soluble forms can also be degraded by CMA or the UPS. The long-standing question whether it is the large aggregate which is degraded wholesale or if it is dismantled into smaller aggregates that are engulfed by forming autophagosomes still remains unanswered.

## 8. Dysfunction of Autophagy in Proteinopathies

Macroautophagic stress indicates a situation when the normal flow of autophagic degradation is impaired [180]. If macroautophagic stress is caused by defects in protein degradation pathways, the effect is impaired degradation of misfolded proteins and accumulation of protein aggregates. But often, the first indication that autophagy is affected in neurodegenerative diseases and disease models is an abnormal number of autophagosomes and/or amphisomes (fusion product of autophagosomes and late endosomes) (reviewed in [181]). In this case, the defect in autophagy is caused by impaired endocytosis at the late endosome-lysosome level, inhibition of autophagosome maturation, and/or inhibition of lysosomal degradative functions. The pressure on autophagy is increased during aging, in part because CMA activity declines. This is mainly caused by decreased levels of LAMP-2A at the lysosomal membrane [182]. Autophagosome formation declines during aging due to decreased expression of some of the vital autophagy proteins like ATG8 family proteins and Beclin1 [183, 184]. Aging is associated with increased intracellular oxidative stress leading to increased unfolding of proteins. Combined with a decline in autophagosome maturation and/or lysosomal degradation, this helps explaining the late-onset phenotypes often observed for several of the proteinopathies. Induction of autophagosome formation is often suggested as a solution to the problem of macroautophagic stress, and it has shown promising results in cell culture and in vivo models [39, 185]. But in other cases autophagosome formation is not a solution, since maturation of autophagosomes or lysosomal degradation is impaired. Caution is therefore required before trying to boost autophagy as a therapeutic strategy in neurodegenerative diseases. It is imperative to first determine the main cause of dysfunctional autophagy in the different types of proteinopathies before trying to boost or inhibit autophagy/aggrephagy as a therapy.

### 8.1. Accumulation of p62: Proteinopathies in Liver and Muscle

p62 is present in almost all cytoplasmic and nuclear inclusions found in human diseases [186–189]. In most proteinopathies, another aggregation-prone protein is responsible for the formation of the aggregate, and p62 is recruited later and possibly in response to ubiquitination of the aggregate. An example is polyglutamine inclusions that are formed independently of p62 [190]. However, p62 is together with NBR1 and ALFY important for the formation and degradation of p62 bodies in response to puromycin treatment or starvation of HeLa cells [30, 31, 79]. These structures are highly ubiquitinated and substrates for selective autophagy [28, 31]. p62 is also crucial for the formation of two types of pathogenic aggregates found in chronic liver diseases [188, 191], that is, intracellular hyaline bodies found in hepatocellular carcinoma and Mallory-Denk bodies (MBs) found in alcoholic and nonalcoholic steatohepatitis. Similar to p62 bodies, p62 and ubiquitin are major constituents of these structures, but MBs in addition contain abnormal keratins [191]. Most likely, their formation is initially caused by insufficient degradation of p62 bodies by autophagy. What should be noted is that the level of ALFY in the liver is very low [143], and it is tempting to speculate that the tendency of p62 to form aggregates in hepatocytes is caused by this. Apart from Paget's disease of the bone affecting the skeleton, liver is the only organ where p62 has been shown to play a main role in the formation of protein aggregates in human disease.

Autophagy knock-out studies support the hypothesis that p62 bodies develop into stable aggregates if autophagy is impaired. In mice, tissue-specific knockout of autophagy causes accumulation of p62-containing aggregates in neurons [53, 54, 192], hepatocytes [55], skeletal muscle [51, 52], cardiac muscles [193], pancreatic *β* cells [194, 195], and kidneys [196]. A similar accumulation of ubiquitinated aggregates is seen after knockout of autophagy in flies [184, 197]. Importantly, p62, or the *Drosophila* homologue Ref(2)P, is required for the formation of ubiquitinated protein aggregates under autophagy knock-out conditions, both in cell culture and in vivo [28, 31, 55, 198]. The most likely interpretation is that p62-mediated accumulation of ubiquitinated proteins in p62 bodies results in the formation of aggregates that in the absence of autophagy cannot be degraded [55]. In cells lacking p62, the contents of the aggregates are likely to be degraded by the UPS or CMA and the effect of autophagy inhibition is therefore less pronounced. Blockade of autophagy may in fact inhibit the UPS if the p62 level gets very high because p62 may inhibit substrate delivery to the proteasome [101].

NBR1 colocalizes with p62 in the Mallory-Denk bodies in patients with alcoholic steatohepatitis [30], and may therefore contribute to the formation of aggregates in liver. NBR1 also colocalizes with p62, LC3, and phosphorylated tau in ubiquitinated protein aggregates of sporadic inclusion-body myositis (s-IBM) that is the most common degenerative myopathy associated with aging [199, 200]. Hence, it is very likely that p62 and NBR1 cooperate in clearance of protein aggregates by autophagy.

### 8.2. Proteinopathies in Neurodegeneration

There is very little LC3-II or autophagosomes in healthy neurons, but this is due to a very rapid turnover of autophagosomes [177]. Neurons may therefore be vulnerable to inhibition of the flow of autophagosomes at any step downstream of autophagy formation. Autophagy of proteins is part of the normal function of postmitotic neurons and is constitutively needed. Conditional knockout of autophagy in mice causes neuronal degeneration and accumulation of ubiquitinated protein aggregates [53, 54]. This clearly demonstrates that autophagy delays the onset of neurodegenerative diseases. The major component of inclusions formed in neurodegenerative diseases is often a single protein, and the most common intracellular neuronal proteinopathies are formed by *α*-synuclein, tau, TDP-43 (transactive response DNA-binding protein-43), or a mutated protein with extended polyglutamine repeats (see refs. [81, 145, 181, 201]). Aggregation-prone proteins that are mutated in disease are often used as models to study protein aggregation and aggrephagy. Among the best studied neurodegenerative diseases are the *α*-synucleinopathies caused by aggregation of *α*-synuclein and responsible for diseases like Parkinson's disease (PD) and dementia with Lewy bodies [202]. These diseases are characterized by the aggregation of *α*-synuclein into so-called Lewy bodies (LBs), but PD is also associated with lysosomal dysfunction and mitochondrial dysfunction [180, 203]. The LBs of PD and dementia with LBs are likely the disease-associated aggregates morphologically most similar to a “classical” aggresome [204–206]. HDAC6 is a component of LBs, and the formation of LBs depends on ubiquitination of substrates by Parkin and transport mediated by HDAC6 [77, 85]. The *α*-synuclein is degraded by CMA or autophagy [207–209]. In PD and certain tauopathies, there is a block in CMA because accumulation of *α*-synuclein or toxic forms of tau inhibit the CMA translocation complex [207, 210, 211]. Such inhibition of CMA may play a key role in the development of these disorders [181], and it may be responsible for the observed activation of autophagy in PD [209]. Induction of autophagy may have a protective effect on *α*-synuclein-related diseases [212–214]. However, too much autophagy may be toxic if maturation of autophagosomes is impaired. Activation of autophagosome formation may therefore be beneficial at early stages of the disease but may lead to enhanced neuronal degeneration in other settings [180].

Another group of neurodegenerative diseases are 10 different autosomal dominant disorders caused by aggregation of polyglutamine stretches on proteins [201]. These are caused by genes that contain a stretch of repetitive CAG glutamine codons that is unstable and tends to expand. The tendency of polyglutamine stretch containing proteins to aggregate is proportional to the number of glutamine repeats. For Huntington's disease (HD) caused by aggregation of Huntingtin (Htt) fragments, a stretch of around 40 glutamines may be sufficient to cause a disease [201]. Polyglutamine-expanded mutant Htt is degraded by autophagy, and autophagy reduces the toxicity associated with mutant Htt expression both in cell culture and in mouse, fly, and zebrafish models of Huntington's disease [46, 175, 185, 215, 216]. Autophagy also has a role in clearance of other polyglutamine-expanded proteins, including mutant ataxin-3 that is causing the spinocerebellar ataxia type 3 (SCA3) [153]. However, in a fly model of dentatorubral-pallidoluysian atrophy (DRPLA), a disorder caused by mutations in the atrophin-1 protein, autophagy induction was unable to rescue the degenerative phenotype because lysosomal degradation was impaired [217]. There is also evidence that mutant Htt has a negative effect on selective autophagy affecting cargo recognition causing accumulation of “empty” autophagosomes as analyzed by immunoEM [218].

Several neurodegenerative diseases are characterized by inclusions of hyperphosphorylated forms of the microtubule-associated protein tau [219]. The most common and best known disease with tau inclusions is Alzheimer's disease (AD). Tau forms neurofibrillary tangles in AD, but also soluble oligomers of hyperphosphorylated tau contribute to neuronal degeneration [220]. AD is also associated with plaques of amyloid-*β* (*αβ*) peptide produced by cleavage of amyloid precursor protein (APP) by *β*- and *γ*-secretases. In a mouse model, induction of autophagy delayed the onset of AD although it had no effect at later stages associated with formation of plaques and tangles [221]. Tau binds tubulin, and the normal function of tau is to promote stabilization of microtubules in neuronal axons. This is needed for long-distance transport and for the maintenance of cellular morphology. Hyperphosphorylated tau has a reduced affinity for tubulin, and this is believed to result in destabilization of microtubules [219]. As mentioned above, p62 knock-out mice display an AD-like phenotype as they grow older and their brains contain increased amounts of hyperphosphorylated tau and K63-linked ubiquitinated proteins [154, 155].

In a cell model, transport of tau to the aggresome in response to proteasomal inhibition was inhibited by knockdown of HDAC6, and this inhibited clearance of tau aggregates and resulted in an accumulation of insoluble tau [222]. However, although the level of HDAC6 is elevated in AD brain [223] HDAC6 is not present in neurofibrillary tangles or senile plaque of AD [224]. Tau binds to HDAC6 [223], and is an inhibitor of HDAC6 function [225]. HDAC6 knock-out mice have hyperacetylated tubulin, but they are viable and develop without neurological abnormalities [226]. Consistent with this, increased acetylation of tubulin is also found in brain of AD patients. However, tau also inhibits the role of HDAC6 in aggresome formation [225]. Inhibition of aggresome formation favors the formation of smaller and possibly more toxic aggregates and will also have a negative effect on tau degradation.

IBMPFD caused by mutations in p97/VCP primarily affects muscle, brain, and bone tissue and is characterized by the accumulation of cytoplasmic and nuclear ubiquitinated inclusions [107]. Recently, TDP-43 was shown to play a role in frontotemporal dementia induced by expression of mutants of p97/VCP [227, 228]. In a *Drosophila* model of IBMPFD induced by mutant p97/VCP, an elevated level of TDP-43 is directly responsible for the degeneration [228]. TDP-43-positive inclusions are hallmarks of frontotemporal dementia and amyotrophic lateral sclerosis (ALS), and there seems to be a lack of HDAC6 in these inclusions [223]. This correlates with the recent finding that TDP-43 binds to HDAC6 mRNA and knockdown of TDP-43 destabilizes HDAC6 mRNA and leads to downregulation of HDAC6 expression. This causes reduced aggregate formation and increased cytotoxicity in cells expressing a polyQ-expanded ataxin-3 mutant [229]. A novel surprising finding is that TDP-43 appears to stabilize ATG7 mRNA by binding to it via its RRM1 domain. Depletion of TDP-43 caused reduction of the ATG7 mRNA/protein and inhibition of autophagy leading to accumulation of polyubiquitinated proteins and p62 [230]. Hence, functional TDP-43 is important for efficient autophagy.

The reason why IBMPFD mutants of p97/VCP cause accumulation of TDP-43 is not known, but it suggests a role for p97/VCP in degradation of TDP-43 and/or for the segregation of TDP-43 from the ribonucleoprotein particle complex during translation [228]. Mutations in p97/VCP can also cause familial ALS [231]. Very recently, p62 mutations were reported in familial and sporadic ALS patients with 8-9 missense mutations predicted by in silico analyses as candidate disease mutations [232].

### 8.3. Serpinopathies with ER Luminal Location

Similar to the nucleus, the ER is a compartment lacking autophagosomes. However, unlike nuclear aggregates that are not efficiently degraded by autophagy [49], ER luminal aggregates can be degraded by autophagy. Serpinopathies are a group of diseases associated with aggregation of serpin family proteins in ER (reviewed in [233]). Serpins are inhibitors of extra- and intracellular proteases, and they act as pseudosubstrates that upon cleavage change conformation resulting in the formation of an inactive serpin-protease complex. Functional serpins are monomeric. In contrast, mutated variants are associated with the formation of long and ordered polymers caused by the insertion of the flexible and reactive centre loop of one molecule into a *β*-sheet of another. These aggregates cannot be degraded by ERAD and accumulate inside the ER lumen. Aggregation-prone and disease-causing mutant variants are known for several serpin family members, including *α*1-antitrypsin, neuroserpin, *α*1-antichymotrypsin, C1-inhibitor, and antithrombin.

The Z variant of *α*1-antitrypsin forms polymers that accumulate in the ER of hepatocytes, and homozygosity for this mutant allele causes the genetic disease *α*1-antitrypsin deficiency. Since polymerization of serpin mutants occurs posttranslation and most likely after complete folding of the monomers [234], there is a window when monomers can be degraded by ERAD. Hence, the Z-variant of *α*1-antitrypsin is degraded by ERAD, but it also accumulates in autophagosomes in liver cells of patients with *α*1-antitrypsin deficiency and in cell culture [235, 236]. Its degradation is reduced in autophagy deficient cells, and this supports a role for autophagy in degradation of mutated *α*1-antitrypsin [235].

Another familial dementia is FENIB (Familial Encephalopathy with Neuroserpin Inclusion Bodies) that is caused by polymerization of mutant neuroserpin in ER of neurons. Studies of mammalian cells and a *Drosophila* model of serpinopathy revealed that ERAD and macroautophagy cooperate also in degradation of mutant neuroserpin [234]. Autophagic degradation of polymeric neuroserpin and other serpinopathies is probably coupled to autophagic degradation of ER itself. In this process, portions of ER are believed to be engulfed along with proteins and protein aggregates. It remains to be shown whether there exist mechanisms for the specific delivery of serpin polymers to those regions of ER that undergo degradation. No selectivity towards mutated neuroserpin was observed for the autophagic degradation of neuroserpin in neuronal-like PC12 cells, suggesting that degradation of neuroserpin by autophagy is mainly a non-selective bulk degradation process [234].

## 9. Concluding Remarks

Selective autophagy of protein aggregates has emerged as an important protein quality control system in cells, and the last decade has provided some major leaps in our understanding of aggrephagy. The autophagy receptors p62 and NBR1 and the large adaptor protein ALFY play major roles in aggrephagy. The level of ALFY in the brain is high [143], and loss of ALFY or p62 is associated with neurodegeneration [144, 155]. It is anticipated that more autophagy receptors are involved in aggrephagy, and optineurin is one of them. How much can be learned from studies of selective autophagy of intracellular bacteria (xenophagy) that is also relevant for aggrephagy? Novel autophagy receptors like NDP52 and optineurin have emerged from studies of xenophagy, and ubiquitination is heavily involved [26, 134]. Likely, also the selective removal of damaged mitochondria (mitophagy) may provide knowledge applicable to the understanding of aggrephagy. For instance, p62 is involved in clustering of mitochondria during mitophagy [237, 238].

As reflected in this paper, there is recent progress in the understanding of the roles played by chaperones and their cofactors in sorting of misfolded proteins to the different degradation pathways. Chaperones and co-chaperones, particularly BAG3, in addition to p97/VCP, HDAC6, TDP-43, and ubiquilin-1, are important players in the formation of aggregates, and they also affect aggrephagy at several steps. However, the study of autophagic degradation of protein aggregates is still in its infancy in the sense that some fundamental questions remain unanswered. For example, we still do not know what size(s) of aggregates can be degraded by selective autophagy. Is there an upper size limit? Is the most efficient degradation of a large aggregate a combination of UPS-, CMA-, and aggrephagy-mediated degradations? An important role for chaperones and cofactors may then be to orchestrate the different degradative pathways and to help to dissolve the aggregates. There is clearly some confusion in the field as to what are the similarities and differences between different types of protein aggregates described in the literature. How should the different types of aggregates and protein inclusions be classified?

 A central question is whether modulation of aggrephagy is a relevant therapeutic strategy for neurodegenerative diseases and other proteinopathies. There is a direct parallel here to cancer where many clinical trials are under way to test effects of inhibiting or boosting autophagy as part of treatment regimens for various cancers. It may very well be that the broad preliminary conclusion is the same for cancer and neurodegenerative diseases; autophagy is generally acting protectively before advanced disease, while it may be harmful to stimulate autophagy in advanced disease states. In cancer, successful tumor cells often depend on autophagy (so inhibition is the best strategy), and in neurodegenerative diseases there is often already a dysfunctional downstream step so that stimulation of autophagosome formation may not be beneficial. The challenge is now to gain more knowledge about the mechanisms involved in aggrephagy and of the particular deficiencies in these mechanisms that are decisive for onset and progression of neurodegenerative diseases.

## References

[1] Dobson CM (2003). Protein folding and misfolding. *Nature*.

[2] Komander D (2009). The emerging complexity of protein ubiquitination. *Biochemical Society Transactions*.

[3] Ikeda F, Dikic I (2008). Atypical ubiquitin chains: new molecular signals. “protein modifications: beyond the usual suspects” review series. *EMBO Reports*.

[4] Wong E, Cuervo AM (2010). Integration of clearance mechanisms: the proteasome and autophagy. *Cold Spring Harbor Perspectives in Biology*.

[5] Hershko A, Heller H, Elias S, Ciechanover A (1983). Components of ubiquitin-protein ligase system. Resolution, affinity purification, and role in protein breakdown. *Journal of Biological Chemistry*.

[6] Pickart CM (2001). Mechanisms underlying ubiquitination. *Annual Review of Biochemistry*.

[7] Schulman BA, Harper JW (2009). Ubiquitin-like protein activation by E1 enzymes: the apex for downstream signalling pathways. *Nature Reviews Molecular Cell Biology*.

[8] Van Wijk SJ, Timmers HT (2010). The family of ubiquitin-conjugating enzymes (E2s): deciding between life and death of proteins. *The FASEB Journal*.

[9] Nagy V, Dikic I (2010). Ubiquitin ligase complexes: from substrate selectivity to conjugational specificity. *Biological Chemistry*.

[10] Marques AJ, Palanimurugan R, Mafias AC, Ramos PC, Dohmen RJ (2009). Catalytic mechanism and assembly of the proteasome. *Chemical Reviews*.

[11] Tanaka K (2009). The proteasome: overview of structure and functions. *Proceedings of the Japan Academy B*.

[12] Förster F, Lasker K, Nickell S, Sali A, Baumeister W (2010). Toward an integrated structural model of the 26S proteasome. *Molecular and Cellular Proteomics*.

[13] Finley D (2009). Recognition and processing of ubiquitin-protein conjugates by the proteasome. *Annual Review of Biochemistry*.

[14] Mizushima N (2007). Autophagy: process and function. *Genes and Development*.

[15] Mizushima N, Levine B, Cuervo AM, Klionsky DJ (2008). Autophagy fights disease through cellular self-digestion. *Nature*.

[16] Nakatogawa H, Suzuki K, Kamada Y, Ohsumi Y (2009). Dynamics and diversity in autophagy mechanisms: lessons from yeast. *Nature Reviews Molecular Cell Biology*.

[17] Mijaljica D, Prescott M, Devenish RJ (2011). Microautophagy in mammalian cells: revisiting a 40-year-old conundrum. *Autophagy*.

[18] Dice JF (2007). Chaperone-mediated autophagy. *Autophagy*.

[19] Kon M, Cuervo AM (2010). Chaperone-mediated autophagy in health and disease. *FEBS Letters*.

[20] Weidberg H, Shvets E, Elazar Z (2011). Biogenesis and cargo selectivity of autophagosomes. *Annual Review of Biochemistry*.

[21] Xie Z, Klionsky DJ (2007). Autophagosome formation: core machinery and adaptations. *Nature Cell Biology*.

[22] Shpilka T, Weidberg H, Pietrokovski S, Elazar Z (2011). Atg8: an autophagy-related ubiquitin-like protein family. *Genome Biology*.

[23] Nakatogawa H, Ichimura Y, Ohsumi Y (2007). Atg8, a ubiquitin-like protein required for autophagosome formation, mediates membrane tethering and hemifusion. *Cell*.

[24] Kabeya Y, Mizushima N, Yamamoto A, Oshitani-Okamoto S, Ohsumi Y, Yoshimori T (2004). LC3, GABARAP and GATE16 localize to autophagosomal membrane depending on form-II formation. *Journal of Cell Science*.

[25] Kraft C, Peter M, Hofmann K (2010). Selective autophagy: ubiquitin-mediated recognition and beyond. *Nature Cell Biology*.

[26] Johansen T, Lamark T (2011). Selective autophagy mediated by autophagic adapter proteins. *Autophagy*.

[27] Kirkin V, McEwan DG, Novak I, Dikic I (2009). A Role for Ubiquitin in Selective Autophagy. *Molecular Cell*.

[28] Bjørkøy G, Lamark T, Brech A (2005). p62/SQSTM1 forms protein aggregates degraded by autophagy and has a protective effect on huntingtin-induced cell death. *Journal of Cell Biology*.

[29] Ichimura Y, Kumanomidou T, Sou YS (2008). Structural basis for sorting mechanism of p62 in selective autophagy. *Journal of Biological Chemistry*.

[30] Kirkin V, Lamark T, Sou YS (2009). A role for NBR1 in autophagosomal degradation of ubiquitinated substrates. *Molecular Cell*.

[31] Pankiv S, Clausen TH, Lamark T (2007). p62/SQSTM1 binds directly to Atg8/LC3 to facilitate degradation of ubiquitinated protein aggregates by autophagy. *Journal of Biological Chemistry*.

[32] Deretic V (2010). Autophagy in infection. *Current Opinion in Cell Biology*.

[33] Arrasate M, Mitra S, Schweitzer ES, Segal MR, Finkbeiner S (2004). Inclusion body formation reduces levels of mutant huntingtin and the risk of neuronal death. *Nature*.

[34] Kopito RR (2000). Aggresomes, inclusion bodies and protein aggregation. *Trends in Cell Biology*.

[35] Caughey B, Lansbury PT (2003). Protofibrils, pores, fibrils, and neurodegeneration: separating the responsible protein aggregates from the innocent bystanders. *Annual Review of Neuroscience*.

[36] Chiti F, Dobson CM (2006). Protein misfolding, functional amyloid, and human disease. *Annual Review of Biochemistry*.

[37] Ortega Z, Díaz-Hernández M, Maynard CJ, Hernández F, Dantuma NP, Lucas JJ (2010). Acute polyglutamine expression in inducible mouse model unravels ubiquitin/proteasome system impairment and permanent recovery attributable to aggregate formation. *Journal of Neuroscience*.

[38] Zhang X, Qian S-B (2011). Chaperone-mediated hierarchical control in targeting misfolded proteins to aggresomes. *Molecular Biology of the Cell*.

[39] Rubinsztein DC (2006). The roles of intracellular protein-degradation pathways in neurodegeneration. *Nature*.

[40] Itakura E, Mizushima N (2011). p62 targeting to the autophagosome formation site requires self-oligomerization but not LC3 binding. *Journal of Cell Biology*.

[41] Øverbye A, Fengsrud M, Seglen PO (2007). Proteomic analysis of membrane-associated proteins from rat liver autophagosomes. *Autophagy*.

[42] Maattanen P, Gehring K, Bergeron JJM, Thomas DY (2010). Protein quality control in the ER: the recognition of misfolded proteins. *Seminars in Cell and Developmental Biology*.

[43] Wang Q, Liu Y, Soetandyo N, Baek K, Hegde R, Ye Y (2011). A ubiquitin ligase-associated chaperone holdase maintains polypeptides in soluble states for proteasome degradation. *Molecular Cell*.

[44] Pratt WB, Morishima Y, Peng HM, Osawa Y (2010). Proposal for a role of the Hsp90/Hsp70-based chaperone machinery in making triage decisions when proteins undergo oxidative and toxic damage. *Experimental Biology and Medicine*.

[45] Lamark T, Johansen T (2010). Autophagy: links with the proteasome. *Current Opinion in Cell Biology*.

[46] Pandey UB, Nie Z, Batlevi Y (2007). HDAC6 rescues neurodegeneration and provides an essential link between autophagy and the UPS. *Nature*.

[47] Massey AC, Kaushik S, Sovak G, Kiffin R, Cuervo AM (2006). Consequences of the selective blockage of chaperone-mediated autophagy. *Proceedings of the National Academy of Sciences of the United States of America*.

[48] Ding Q, Dimayuga E, Martin S (2003). Characterization of chronic low-level proteasome inhibition on neural homeostasis. *Journal of Neurochemistry*.

[49] Iwata A, Christianson JC, Bucci M (2005). Increased susceptibility of cytoplasmic over nuclear polyglutamine aggregates to autophagic degradation. *Proceedings of the National Academy of Sciences of the United States of America*.

[50] Kaushik S, Massey AC, Mizushima N, Cuervo AM (2008). Constitutive activation of chaperone-mediated autophagy in cells with impaired macroautophagy. *Molecular Biology of the Cell*.

[51] Masiero E, Agatea L, Mammucari C (2009). Autophagy is required to maintain muscle mass. *Cell Metabolism*.

[52] Raben N, Hill V, Shea L (2008). Suppression of autophagy in skeletal muscle uncovers the accumulation of ubiquitinated proteins and their potential role in muscle damage in Pompe disease. *Human Molecular Genetics*.

[53] Hara T, Nakamura K, Matsui M (2006). Suppression of basal autophagy in neural cells causes neurodegenerative disease in mice. *Nature*.

[54] Komatsu M, Waguri S, Chiba T (2006). Loss of autophagy in the central nervous system causes neurodegeneration in mice. *Nature*.

[55] Komatsu M, Waguri S, Koike M (2007). Homeostatic levels of p62 control cytoplasmic inclusion body formation in autophagy-deficient mice. *Cell*.

[56] Dice JF (1990). Peptide sequences that target cytosolic proteins for lysosomal proteolysis. *Trends in Biochemical Sciences*.

[57] Kiffin R, Christian C, Knecht E, Cuervo AM (2004). Activation of chaperone-mediated autophagy during oxidative stress. *Molecular Biology of the Cell*.

[58] Ballinger CA, Connell P, Wu Y (1999). Identification of CHIP, a novel tetratricopeptide repeat-containing protein that interacts with heat shock proteins and negatively regulates chaperone functions. *Molecular and Cellular Biology*.

[59] Connell P, Ballinger CA, Jiang J (2001). The co-chaperone CHIP regulates protein triage decisions mediated by heat-shock proteins. *Nature Cell Biology*.

[60] Meacham GC, Patterson C, Zhang W, Younger JM, Cyr DM (2001). The Hsc70 co-chaperone CHIP targets immature CFTR for proteasomal degradation. *Nature Cell Biology*.

[61] Scaglione KM, Zavodszky E, Todi S (2011). Ube2w and ataxin-3 coordinately regulate the ubiquitin ligase CHIP. *Molecular Cell*.

[62] Kettern N, Dreiseidler M, Tawo R, Höhfeld J (2010). Chaperone-assisted degradation: multiple paths to destruction. *Biological Chemistry*.

[63] Carra S, Seguin SJ, Lambert H, Landry J (2008). HspB8 chaperone activity toward poly(Q)-containing proteins depends on its association with Bag3, a stimulator of macroautophagy. *Journal of Biological Chemistry*.

[64] Crippa V, Sau D, Rusmini P (2010). The small heat shock protein B8 (HspB8) promotes autophagic removal of misfolded proteins involved in amyotrophic lateral sclerosis (ALS). *Human Molecular Genetics*.

[65] Homma S, Iwasaki M, Shelton GD, Engvall E, Reed JC, Takayama S (2006). BAG3 deficiency results in fulminant myopathy and early lethality. *American Journal of Pathology*.

[66] Arndt V, Dick N, Tawo R (2010). Chaperone-assisted selective autophagy is essential for muscle maintenance. *Current Biology*.

[67] Selcen D, Muntoni F, Burton BK (2009). Mutation in BAG3 causes severe dominant childhood muscular dystrophy. *Annals of Neurology*.

[68] Gamerdinger M, Hajieva P, Kaya AM, Wolfrum U, Hartl FU, Behl C (2009). Protein quality control during aging involves recruitment of the macroautophagy pathway by BAG3. *The EMBO Journal*.

[69] Lelouard H, Ferrand V, Marguet D (2004). Dendritic cell aggresome-like induced structures are dedicated areas for ubiquitination and storage of newly synthesized defective proteins. *Journal of Cell Biology*.

[70] Lelouard H, Gatti E, Cappello F, Gresser O, Camosseto V, Pierre P (2002). Transient aggregation of ubiquitinated proteins during dendritic cell maturation. *Nature*.

[71] Szeto J, Kaniuk NA, Canadien V (2006). ALIS are stress-induced protein storage compartments for substrates of the proteasome and autophagy. *Autophagy*.

[72] Kettern N, Rogon C, Limmer A, Schild H, Höhfeld J (2011). The Hsc/Hsp70 co-chaperone network controls antigen aggregation and presentation during maturation of professional antigen presenting cells. *Plos ONE*.

[73] Munz C (2010). Antigen processing via autophagy - not only for MHC class II presentation anymore?. *Current Opinion in Immunology*.

[74] Wenger T, Terawaki S, Camosseto V (2012). Autophagy inhibition promotes defective neosynthesized proteins storage in ALIS, and induces redirection toward proteasome processing and MHCI-restricted presentation. *Autophagy*.

[75] Kaniuk NA, Kiraly M, Bates H, Vranic M, Volchuk A, Brumell JH (2007). Ubiquitinated-protein aggregates form in pancreatic *β*-cells during diabetes-induced oxidative stress and are regulated by autophagy. *Diabetes*.

[76] Johnston JA, Ward CL, Kopito RR (1998). Aggresomes: a cellular response to misfolded proteins. *Journal of Cell Biology*.

[77] Kawaguchi Y, Kovacs JJ, McLaurin A, Vance JM, Ito A, Yao TP (2003). The deacetylase HDAC6 regulates aggresome formation and cell viability in response to misfolded protein stress. *Cell*.

[78] Kaganovich D, Kopito R, Frydman J (2008). Misfolded proteins partition between two distinct quality control compartments. *Nature*.

[79] Clausen TH, Lamark T, Isakson P (2010). p62/SQSTM1 and ALFY interact to facilitate the formation of p62 bodies/ALIS and their degradation by autophagy. *Autophagy*.

[80] Filimonenko M, Isakson P, Finley KD (2010). The selective macroautophagic degradation of aggregated proteins requires the PI3P-binding protein alfy. *Molecular Cell*.

[81] Knævelsrud H, Simonsen A (2010). Fighting disease by selective autophagy of aggregate-prone proteins. *FEBS Letters*.

[82] Iwata A, Riley BE, Johnston JA, Kopito RR (2005). HDAC6 and microtubules are required for autophagic degradation of aggregated Huntingtin. *Journal of Biological Chemistry*.

[83] Yao T (2010). The role of ubiquitin in autophagy-dependent protein aggregate processing. *Genes and Cancer*.

[84] Pan T, Kondo S, Le W, Jankovic J (2008). The role of autophagy-lysosome pathway in neurodegeneration associated with Parkinson’s disease. *Brain*.

[85] Olzmann JA, Li A, Chudaev MV (2007). Parkin-mediated K63-linked polyubiquitination targets misfolded DJ-1 to aggresomes via binding to HDAC6. *Journal of Cell Biology*.

[86] Lee JY, Koga H, Kawaguchi Y (2010). HDAC6 controls autophagosome maturation essential for ubiquitin-selective quality-control autophagy. *The EMBO Journal*.

[87] Gamerdinger M, Kaya AM, Wolfrum U, Clement AM, Behl C (2011). BAG3 mediates chaperone-based aggresome-targeting and selective autophagy of misfolded proteins. *EMBO Reports*.

[88] Yamanaka K, Sasagawa Y, Ogura T (2012). Recent advances in p97/VCP/Cdc48 cellular functions. *Biochimica et Biophysica Acta*.

[89] Dargemont C, Ossareh-Nazari B (2012). Cdc48/p97, a key actor in the interplay between autophagy and ubiquitin/proteasome catabolic pathways. *Biochimica et Biophysica Acta*.

[90] Dalal S, Rosser MFN, Cyr DM, Hanson PI (2004). Distinct roles for the AAA ATPases NSF and p97 in the secretory pathway. *Molecular Biology of the Cell*.

[91] Wójcik C, Rowicka M, Kudlicki A (2006). Valosin-containing protein (p97) is a regulator of endoplasmic reticulum stress and of the degradation of N-end rule and ubiquitin-fusion degradation pathway substrates in mammalian cells. *Molecular Biology of the Cell*.

[92] Wójcik C, Yano M, DeMartino GN (2004). RNA interference of valosin-containing protein (VCP/p97) reveals multiple cellular roles linked to ubiquitin/proteasome-dependent proteolysis. *Journal of Cell Science*.

[93] Pye VE, Dreveny I, Briggs LC (2006). Going through the motions: the ATPase cycle of p97. *Journal of Structural Biology*.

[94] Braun S, Matuschewski K, Rape M, Thoms S, Jentsch S (2002). Role of the ubiquitin-selective CDC48UFD1/NPL4 chaperone (segregase) in ERAD of OLE1 and other substrates. *The EMBO Journal*.

[95] Ramadan K, Bruderer R, Spiga FM (2007). Cdc48/p97 promotes reformation of the nucleus by extracting the kinase Aurora B from chromatin. *Nature*.

[96] Rape M, Hoppe T, Gorr I, Kalocay M, Richly H, Jentsch S (2001). Mobilization of processed, membrane-tethered SPT23 transcription factor by CDC48UFD1/NPL4, a ubiquitin-selective chaperone. *Cell*.

[97] Greenblatt EJ, Olzmann JA, Kopito RR (2011). Derlin-1 is a rhomboid pseudoprotease required for the dislocation of mutant *α*-1 antitrypsin from the endoplasmic reticulum. *Nature Structural and Molecular Biology*.

[98] Hirsch C, Gauss R, Horn SC, Neuber O, Sommer T (2009). The ubiquitylation machinery of the endoplasmic reticulum. *Nature*.

[99] Tanaka A, Cleland MM, Xu S (2010). Proteasome and p97 mediate mitophagy and degradation of mitofusins induced by Parkin. *Journal of Cell Biology*.

[100] Xu S, Peng G, Wang Y, Fang S, Karbowsk M (2011). The AAA-ATPase p97 is essential for outer mitochondrial membrane protein turnover. *Molecular Biology of the Cell*.

[101] Korolchuk VI, Mansilla A, Menzies FM, Rubinsztein DC (2009). Autophagy inhibition compromises degradation of ubiquitin-proteasome pathway substrates. *Molecular Cell*.

[102] Ju JS, Miller SE, Hanson PI, Weihl CC (2008). Impaired protein aggregate handling and clearance underlie the pathogenesis of p97/VCP-associated disease. *Journal of Biological Chemistry*.

[103] Kitami MI, Kitami T, Nagahama M (2006). Dominant-negative effect of mutant valosin-containing protein in aggresome formation. *FEBS Letters*.

[104] Kobayashi T, Manno A, Kakizuka A (2007). Involvement of valosin-containing protein (VCP)/p97 in the formation and clearance of abnormal protein aggregates. *Genes to Cells*.

[105] Boyault C, Gilquin B, Zhang Y (2006). HDAC6-p97/VCP controlled polyubiquitin chain turnover. *EMBO Journal*.

[106] Tresse E, Salomons FA, Vesa J (2010). VCP/p97 is essential for maturation of ubiquitin-containing autophagosomes and this function is impaired by mutations that cause IBMPFD. *Autophagy*.

[107] Ju JS, Weihl CC (2010). Inclusion body myopathy, Paget’s disease of the bone and fronto-temporal dementia: a disorder of autophagy. *Human Molecular Genetics*.

[108] Tang WK, Li D, Li CC (2010). A novel ATP-dependent conformation in p97 N-D1 fragment revealed by crystal structures of disease-related mutants. *The EMBO Journal*.

[109] Fernández-Sáiz V, Buchberger A (2010). Imbalances in p97 co-factor interactions in human proteinopathy. *EMBO Reports*.

[110] Weihl CC, Dalal S, Pestronk A, Hanson PI (2006). Inclusion body myopathy-associated mutations in p97/VCP impair endoplasmic reticulum-associated degradation. *Human Molecular Genetics*.

[111] Weihl CC, Miller SE, Hanson PI, Pestronk A (2007). Transgenic expression of inclusion body myopathy associated mutant p97/VCP causes weakness and ubiquitinated protein inclusions in mice. *Human Molecular Genetics*.

[112] Buchberger A (2002). From UBA to UBX: new words in the ubiquitin vocabulary. *Trends in Cell Biology*.

[113] Walters KJ, Kleijnen MF, Goh AM, Wagner G, Howley PM (2002). Structural studies of the interaction between ubiquitin family proteins and proteasome subunit S5a. *Biochemistry*.

[114] Lim PJ, Danner R, Liang J (2009). Ubiquilin and p97/VCP bind erasin, forming a complex involved in ERAD. *Journal of Cell Biology*.

[115] Rothenberg C, Srinivasan D, Mah L (2010). Ubiquilin functions in autophagy and is degraded by chaperone-mediated autophagy. *Human Molecular Genetics*.

[116] Heir R, Ablasou C, Dumontier E, Elliott M, Fagotto-Kaufmann C, Bedford FK (2006). The UBL domain of PLIC-1 regulates aggresome formation. *EMBO Reports*.

[117] Massey LK, Mah AL, Ford DL (2004). Overexpression of ubiquilin decreases ubiquitination and degradation of presenilin proteins. *Journal of Alzheimer’s Disease*.

[118] Regan-Klapisz E, Sorokina I, Voortman J (2005). Ubiquilin recruits Eps15 into ubiquitin-rich cytoplasmic aggregates via a UIM-UBL interaction. *Journal of Cell Science*.

[119] Wang H, Lim PJ, Yin C, Rieckher M, Vogel BE, Monteiro MJ (2006). Suppression of polyglutamine-induced toxicity in cell and animal models of Huntington’s disease by ubiquilin. *Human Molecular Genetics*.

[120] Wang H, Monteiro MJ (2007). Ubiquilin interacts and enhances the degradation of expanded-polyglutamine proteins. *Biochemical and Biophysical Research Communications*.

[121] N’Diaye EN, Debnath J, Brown EJ (2009). Ubiquilins accelerate autophagosome maturation and promote cell survival during nutrient starvation. *Autophagy*.

[122] N’Diaye EN, Kajihara KK, Hsieh I, Morisaki H, Debnath J, Brown EJ (2009). PLIC proteins or ubiquilins regulate autophagy-dependent cell survival during nutrient starvation. *EMBO Reports*.

[123] Stieren ES, El Ayadi A, Xiao Y (2011). Ubiquilin-1 is a molecular chaperone for the amyloid precursor protein. *Journal of Biological Chemistry*.

[124] Lamark T, Perander M, Outzen H (2003). Interaction codes within the family of mammalian phox and bem1p domain-containing proteins. *Journal of Biological Chemistry*.

[125] Noda NN, Kumeta H, Nakatogawa H (2008). Structural basis of target recognition by Atg8/LC3 during selective autophagy. *Genes to Cells*.

[126] Rozenknop A, Rogov VV, Rogova NY (2011). Characterization of the interaction of GABARAPL-1 with the LIR motif of NBR1. *Journal of Molecular Biology*.

[127] Noda NN, Ohsumi Y, Inagaki F (2010). Atg8-family interacting motif crucial for selective autophagy. *FEBS Letters*.

[128] Wilson MI, Gill DJ, Perisic O, Quinn MT, Williams RL (2003). PB1 domain-mediated heterodimerization in NADPH oxidase and signaling complexes of atypical protein kinase C with Par6 and p62. *Molecular Cell*.

[129] Svenning S, Lamark T, Krause K, Johansen T (2011). Plant NBR1 is a selective autophagy substrate and a functional hybrid of the mammalian autophagic adapters NBR1 and p62/SQSTM1. *Autophagy*.

[130] Kanki T, Wang K, Cao Y, Baba M, Klionsky DJ (2009). Atg32 is a mitochondrial protein that confers selectivity during mitophagy. *Developmental Cell*.

[131] Okamoto K, Kondo-Okamoto N, Ohsumi Y (2009). Mitochondria-anchored receptor Atg32 mediates degradation of mitochondria via selective autophagy. *Developmental Cell*.

[132] Novak I, Kirkin V, McEwan DG (2010). Nix is a selective autophagy receptor for mitochondrial clearance. *EMBO Reports*.

[133] Thurston TL, Ryzhakov G, Bloor S, von Muhlinen N, Randow F (2009). The TBK1 adaptor and autophagy receptor NDP52 restricts the proliferation of ubiquitin-coated bacteria. *Nature Immunology*.

[134] Wild P, Farhan H, McEwan DG (2011). Phosphorylation of the autophagy receptor optineurin restricts Salmonella growth. *Science*.

[135] Jiang S, Wells CD, Roach PJ (2011). Starch-binding domain-containing protein 1 (Stbd1) and glycogen metabolism: identification of the Atg8 family interacting motif (AIM) in Stbd1 required for interaction with GABARAPL1. *Biochemical and Biophysical Research Communications*.

[136] Gal J, Ström AL, Kwinter DM (2009). Sequestosome 1/p62 links familial ALS mutant SOD1 to LC3 via an ubiquitin-independent mechanism. *Journal of Neurochemistry*.

[137] Watanabe Y, Tanaka M (2011). p62/SQSTM1 in autophagic clearance of a non-ubiquitylated substrate. *Journal of Cell Science*.

[138] Orvedahl A, MacPherson S, Sumpter R, Tallóczy Z, Zou Z, Levine B (2010). Autophagy protects against sindbis virus infection of the central nervous system. *Cell Host and Microbe*.

[139] Zhang Y, Yan L, Zhou Z (2009). SEPA-1 mediates the specific recognition and degradation of P granule components by autophagy in C. elegans. *Cell*.

[140] Kim PK, Hailey DW, Mullen RT, Lippincott-Schwartz J (2008). Ubiquitin signals autophagic degradation of cytosolic proteins and peroxisomes. *Proceedings of the National Academy of Sciences of the United States of America*.

[141] Pohl C, Jentsch S (2009). Midbody ring disposal by autophagy is a post-abscission event of cytokinesis. *Nature Cell Biology*.

[142] Kuo TC, Chen CT, Baron D (2011). Midbody accumulation through evasion of autophagy contributes to cellular reprogramming and tumorigenicity. *Nature Cell Biology*.

[143] Simonsen A, Birkeland HC, Gillooly DJ (2004). Alfy, a novel FYVE-domain-containing protein associated with protein granules and autophagic membranes. *Journal of Cell Science*.

[144] Finley KD, Edeen PT, Cumming RC (2003). Blue cheese mutations define a novel, conserved gene involved in progressive neural degeneration. *Journal of Neuroscience*.

[145] Yamamoto A, Simonsen A (2011). The elimination of accumulated and aggregated proteins: a role for aggrephagy in neurodegeneration. *Neurobiology of Disease*.

[146] McEwan DG, Dikic I (2011). The three musketeers of autophagy: phosphorylation, ubiquitylation and acetylation. *Trends in Cell Biology*.

[147] Tan JM, Wong ES, Dawson VL, Dawson TM, Lim KL (2008). Lysine 63-linked polyubiquitin potentially partners with p62 to promote the clearance of protein inclusions by autophagy. *Autophagy*.

[148] Tan JM, Wong ES, Kirkpatrick DS (2008). Lysine 63-linked ubiquitination promotes the formation and autophagic clearance of protein inclusions associated with neurodegenerative diseases. *Human Molecular Genetics*.

[149] Seibenhener ML, Babu JR, Geetha T, Wong HC, Krishna NR, Wooten MW (2004). Sequestosome 1/p62 is a polyubiquitin chain binding protein involved in ubiquitin proteasome degradation. *Molecular and Cellular Biology*.

[150] Moscat J, Diaz-Meco MT, Wooten MW (2007). Signal integration and diversification through the p62 scaffold protein. *Trends in Biochemical Sciences*.

[151] Burnett BG, Pittman RN (2005). The polyglutamine neurodegenerative protein ataxin 3 regulates aggresome formation. *Proceedings of the National Academy of Sciences of the United States of America*.

[152] Winborn BJ, Travis SM, Todi SV (2008). The deubiquitinating enzyme ataxin-3, a polyglutamine disease protein, edits Lys63 linkages in mixed linkage ubiquitin chains. *Journal of Biological Chemistry*.

[153] Menzies FM, Huebener J, Renna M, Bonin M, Riess O, Rubinsztein DC (2010). Autophagy induction reduces mutant ataxin-3 levels and toxicity in a mouse model of spinocerebellar ataxia type 3. *Brain*.

[154] Wooten MW, Geetha T, Babu JR (2008). Essential role of sequestosome 1/p62 in regulating accumulation of Lys63-ubiquitinated proteins. *Journal of Biological Chemistry*.

[155] Ramesh Babu J, Lamar Seibenhener M, Peng J (2008). Genetic inactivation of p62 leads to accumulation of hyperphosphorylated tau and neurodegeneration. *Journal of Neurochemistry*.

[156] Zucchelli S, Codrich M, Marcuzzi F (2010). TRAF6 promotes atypical ubiquitination of mutant DJ-1 and alpha-synuclein and is localized to Lewy bodies in sporadic Parkinson’s disease brains. *Human Molecular Genetics*.

[157] Shi CS, Kehrl JH (2010). TRAF6 and A20 regulate lysine 63-linked ubiquitination of Beclin-1 to control TLR4-induced Autophagy. *Science Signaling*.

[158] Cherra SJ, Kulich SM, Uechi G (2010). Regulation of the autophagy protein LC3 by phosphorylation. *Journal of Cell Biology*.

[159] Matsumoto G, Wada K, Okuno M, Kurosawa M, Nukina N (2011). Serine 403 phosphorylation of p62/SQSTM1 regulates selective autophagic clearance of ubiquitinated proteins. *Molecular Cell*.

[160] Maruyama H, Morino H, Ito H (2010). Mutations of optineurin in amyotrophic lateral sclerosis. *Nature*.

[161] Osawa T, Mizuno Y, Fujita Y, Takatama M, Nakazato Y, Okamoto K (2011). Optineurin in neurodegenerative diseases. *Neuropathology*.

[162] Schwab C, Yu S, McGeer EG, McGeer PL (2012). Optineurin in Huntington's disease intranuclear inclusions. *Neuroscience Letters*.

[163] van Blitterswijk M, van Vught PW, van Es MA Novel optineurin mutations in sporadic amyotrophic lateral sclerosis patients.

[164] Lee IH, Finkel T (2009). Regulation of autophagy by the p300 acetyltransferase. *Journal of Biological Chemistry*.

[165] Jeong H, Then F, Melia TJ (2009). Acetylation Targets Mutant Huntingtin to Autophagosomes for Degradation. *Cell*.

[166] Mookerjee S, Papanikolaou T, Guyenet SJ (2009). Posttranslational modification of ataxin-7 at lysine 257 prevents autophagy-mediated turnover of an N-terminal caspase-7 cleavage fragment. *Journal of Neuroscience*.

[167] Fan W, Tang Z, Chen D (2010). Keap1 facilitates p62-mediated ubiquitin aggregate clearance via autophagy. *Autophagy*.

[168] Jain A, Lamark T, Sjøttem E (2010). p62/SQSTM1 is a target gene for transcription factor NRF2 and creates a positive feedback loop by inducing antioxidant response element-driven gene transcription. *Journal of Biological Chemistry*.

[169] Komatsu M, Kurokawa H, Waguri S (2010). The selective autophagy substrate p62 activates the stress responsive transcription factor Nrf2 through inactivation of Keap1. *Nature Cell Biology*.

[170] Lau A, Wang XJ, Zhao F (2010). A noncanonical mechanism of Nrf2 activation by autophagy deficiency: direct interaction between keap1 and p62. *Molecular and Cellular Biology*.

[171] Nakaso K, Nakamura C, Sato H, Imamura K, Takeshima T, Nakashima K (2006). Novel cytoprotective mechanism of anti-parkinsonian drug deprenyl: PI3K and Nrf2-derived induction of antioxidative proteins. *Biochemical and Biophysical Research Communications*.

[172] Nakaso K, Yoshimoto Y, Nakano T (2004). Transcriptional activation of p62/A170/ZIP during the formation of the aggregates: possible mechanisms and the role in Lewy body formation in Parkinson’s disease. *Brain Research*.

[173] Pankiv S, Lamark T, Bruun JA, Øvervatn A, Bjørkøy G, Johansen T (2010). Nucleocytoplasmic shuttling of p62/SQSTM1 and its role in recruitment of nuclear polyubiquitinated proteins to promyelocytic leukemia bodies. *Journal of Biological Chemistry*.

[174] Boland B, Nixon RA (2006). Neuronal macroautophagy: from development to degeneration. *Molecular Aspects of Medicine*.

[175] Ravikumar B, Duden R, Rubinsztein DC (2002). Aggregate-prone proteins with polyglutamine and polyalanine expansions are degraded by autophagy. *Human Molecular Genetics*.

[176] Yamamoto A, Cremona ML, Rothman JE (2006). Autophagy-mediated clearance of huntingtin aggregates triggered by the insulin-signaling pathway. *Journal of Cell Biology*.

[177] Boland B, Kumar A, Lee S (2008). Autophagy induction and autophagosome clearance in neurons: relationship to autophagic pathology in Alzheimer’s disease. *Journal of Neuroscience*.

[178] Doyle SM, Wickner S (2009). Hsp104 and ClpB: protein disaggregating machines. *Trends in Biochemical Sciences*.

[179] Shorter J (2011). The mammalian disaggregase machinery: Hsp110 synergizes with Hsp70 and Hsp40 to catalyze protein disaggregation and reactivation in a cell-free system. *Plos ONE*.

[180] Xilouri M, Stefanis L (2011). Autophagic pathways in Parkinson disease and related disorders. *Expert Reviews in Molecular Medicine*.

[181] Wong E, Cuervo AM (2010). Autophagy gone awry in neurodegenerative diseases. *Nature Neuroscience*.

[182] Cuervo AM, Dice JF (2000). Age-related decline in chaperone-mediated autophagy. *Journal of Biological Chemistry*.

[183] Shibata M, Lu T, Furuya T (2006). Regulation of intracellular accumulation of mutant huntingtin by beclin 1. *Journal of Biological Chemistry*.

[184] Simonsen A, Cumming RC, Brech A, Isakson P, Schubert DR, Finley KD (2008). Promoting basal levels of autophagy in the nervous system enhances longevity and oxidant resistance in adult Drosophila. *Autophagy*.

[185] Ravikumar B, Vacher C, Berger Z (2004). Inhibition of mTOR induces autophagy and reduces toxicity of polyglutamine expansions in fly and mouse models of Huntington disease. *Nature Genetics*.

[186] Kuusisto E, Salminen A, Alafuzoff I (2001). Ubiquitin-binding protein p62 is present in neuronal and glial inclusions in human tauopathies and synucleinopathies. *NeuroReport*.

[187] Settembre C, Fraldi A, Jahreiss L (2008). A block of autophagy in lysosomal storage disorders. *Human Molecular Genetics*.

[188] Strnad P, Zatloukal K, Stumptner C, Kulaksiz H, Denk H (2008). Mallory-Denk-bodies: lessons from keratin-containing hepatic inclusion bodies. *Biochimica et Biophysica Acta*.

[189] Zatloukal K, Stumptner C, Fuchsbichler A (2002). p62 is a common component of cytoplasmic inclusions in protein aggregation diseases. *American Journal of Pathology*.

[190] Nagaoka U, Kim K, Nihar RJ (2004). Increased expression of p62 in expanded polyglutamine-expressing cells and its association with polyglutamine inclusions. *Journal of Neurochemistry*.

[191] Denk H, Stumptner C, Fuchsbichler A (2006). Are the Mallory bodies and intracellular hyaline bodies in neoplastic and non-neoplastic hepatocytes related?. *The Journal of Pathology*.

[192] Liang CC, Wang C, Peng X, Gan B, Guan JL (2010). Neural-specific deletion of FIP200 leads to cerebellar degeneration caused by increased neuronal death and axon degeneration. *Journal of Biological Chemistry*.

[193] Nakai A, Yamaguchi O, Takeda T (2007). The role of autophagy in cardiomyocytes in the basal state and in response to hemodynamic stress. *Nature Medicine*.

[194] Ebato C, Uchida T, Arakawa M (2008). Autophagy is important in islet homeostasis and compensatory increase of beta cell mass in response to high-fat diet. *Cell Metabolism*.

[195] Jung HS, Chung KW, Won Kim J (2008). Loss of autophagy diminishes pancreatic *β* cell mass and function with resultant hyperglycemia. *Cell Metabolism*.

[196] Hartleben B, Gödel M, Meyer-Schwesinger C (2010). Autophagy influences glomerular disease susceptibility and maintains podocyte homeostasis in aging mice. *Journal of Clinical Investigation*.

[197] Juhász G, Érdi B, Sass M, Neufeld TP (2007). Atg7-dependent autophagy promotes neuronal health, stress tolerance, and longevity but is dispensable for metamorphosis in Drosophila. *Genes and Development*.

[198] Nezis IP, Simonsen A, Sagona AP (2008). Ref(2)P, the Drosophila melanogaster homologue of mammalian p62, is required for the formation of protein aggregates in adult brain. *Journal of Cell Biology*.

[199] D'Agostino C, Nogalska A, Cacciottolo M, King Engel W, Askanas V (2011). Abnormalities of NBR1, a novel autophagy-associated protein, in muscle fibers of sporadic inclusion-body myositis. *Acta Neuropathologica*.

[200] Nogalska A, Terracciano C, D’Agostino C, King Engel W, Askanas V (2009). p62/SQSTM1 is overexpressed and prominently accumulated in inclusions of sporadic inclusion-body myositis muscle fibers, and can help differentiating it from polymyositis and dermatomyositis. *Acta Neuropathologica*.

[201] Jimenez-Sanchez M, Thomson F, Zavodszky E, Rubinsztein DC Autophagy and polyglutamine diseases.

[202] Spillantini MG, Crowther RA, Jakes R, Hasegawa M, Goedert M (1998). *α*-Synuclein in filamentous inclusions of Lewy bodies from Parkinson’s disease and dementia with Lewy bodies. *Proceedings of the National Academy of Sciences of the United States of America*.

[203] Narendra D, Tanaka A, Suen DF, Youle RJ (2008). Parkin is recruited selectively to impaired mitochondria and promotes their autophagy. *Journal of Cell Biology*.

[204] Ardley HC, Scott GB, Rose SA, Tan NGS, Markham AF, Robinson PA (2003). Inhibition of proteasomal activity causes inclusion formation in neuronal and non-neuronal cells overexpressing parkin. *Molecular Biology of the Cell*.

[205] Olanow CW, Perl DP, DeMartino GN, McNaught KSP (2004). Lewy-body formation is an aggresome-related process: a hypothesis. *Lancet Neurology*.

[206] Tanaka M, Kim YM, Lee G, Junn E, Iwatsubo T, Mouradian MM (2004). Aggresomes formed by *α*-synuclein and synphilin-1 are cytoprotective. *Journal of Biological Chemistry*.

[207] Cuervo AM, Stafanis L, Fredenburg R, Lansbury PT, Sulzer D (2004). Impaired degradation of mutant *α*-synuclein by chaperone-mediated autophagy. *Science*.

[208] Lee HJ, Khoshaghideh F, Patel S, Lee SJ (2004). Clearance of *α*-synuclein oligomeric intermediates via the lysosomal degradation pathway. *Journal of Neuroscience*.

[209] Webb JL, Ravikumar B, Atkins J, Skepper JN, Rubinsztein DC (2003). *α*-synuclein is degraded by both autophagy and the proteasome. *Journal of Biological Chemistry*.

[210] Martinez-Vicente M, Talloczy Z, Kaushik S (2008). Dopamine-modified *α*-synuclein blocks chaperone-mediated autophagy. *Journal of Clinical Investigation*.

[211] Wang Y, Martinez-Vicente M, Kruger U (2009). Tau fragmentation, aggregation and clearance: the dual role of lysosomal processing. *Human Molecular Genetics*.

[212] Crews L, Spencer B, Desplats P (2010). Selective molecular alterations in the autophagy pathway in patients with lewy body disease and in models of *α*-synucleinopathy. *Plos ONE*.

[213] Spencer B, Potkar R, Trejo M (2009). Beclin 1 gene transfer activates autophagy and ameliorates the neurodegenerative pathology in *α*-synuclein models of Parkinson’s and Lewy body diseases. *Journal of Neuroscience*.

[214] Yu WH, Dorado B, Figueroa HY (2009). Metabolic activity determines efficacy of macroautophagic clearance of pathological oligomeric *α*-synuclein. *American Journal of Pathology*.

[215] Berger Z, Ravikumar B, Menzies FM (2006). Rapamycin alleviates toxicity of different aggregate-prone proteins. *Human Molecular Genetics*.

[216] Williams A, Sarkar S, Cuddon P (2008). Novel targets for Huntington’s disease in an mTOR-independent autophagy pathway. *Nature Chemical Biology*.

[217] Nisoli I, Chauvin JP, Napoletano F (2010). Neurodegeneration by polyglutamine Atrophin is not rescued by induction of autophagy. *Cell Death and Differentiation*.

[218] Martinez-Vicente M, Talloczy Z, Wong E (2010). Cargo recognition failure is responsible for inefficient autophagy in Huntington’s disease. *Nature Neuroscience*.

[219] Spires-Jones TL, Stoothoff WH, de Calignon A, Jones PB, Hyman BT (2009). Tau pathophysiology in neurodegeneration: a tangled issue. *Trends in Neurosciences*.

[220] Wittmann CW, Wszolek MF, Shulman JM (2001). Tauopathy in Drosophila: neurodegeneration without neurofibrillary tangles. *Science*.

[221] Majumder S, Richardson A, Strong R, Oddo S (2011). Inducing autophagy by rapamycin before, but not after, the formation of plaques and tangles ameliorates cognitive deficits. *Plos ONE*.

[222] Guthrie CR, Kraemer BC (2011). Proteasome inhibition drives HDAC6-dependent recruitment of tau to aggresomes. *Journal of Molecular Neuroscience*.

[223] Ding H, Dolan PJ, Johnson GVW (2008). Histone deacetylase 6 interacts with the microtubule-associated protein tau. *Journal of Neurochemistry*.

[224] Miki Y, Mori F, Tanji K, Kakita A, Takahashi H, Wakabayashi K (2011). Accumulation of histone deacetylase 6, an aggresome-related protein, is specific to Lewy bodies and glial cytoplasmic inclusions. *Neuropathology*.

[225] Perez M, Santa-Maria I, de Barreda EG (2009). Tau—an inhibitor of deacetylase HDAC6 function. *Journal of Neurochemistry*.

[226] Zhang Y, Kwon S, Yamaguchi T (2008). Mice lacking histone deacetylase 6 have hyperacetylated tubulin but are viable and develop normally. *Molecular and Cellular Biology*.

[227] Neumann M, Mackenzie IR, Cairns NJ (2007). TDP-43 in the ubiquitin pathology of frontotemporal dementia with VCP gene mutations. *Journal of Neuropathology and Experimental Neurology*.

[228] Ritson GP, Custer SK, Freibaum BD (2010). TDP-43 mediates degeneration in a novel Drosophila model of disease caused by mutations in VCP/p97. *Journal of Neuroscience*.

[229] Fiesel FC, Voigt A, Weber SS (2010). Knockdown of transactive response DNA-binding protein (TDP-43) downregulates histone deacetylase 6. *The EMBO Journal*.

[230] Bose JK, Huang CC, Shen CK (2011). Regulation of autophagy by neuropathological protein TDP-43. *Journal of Biological Chemistry*.

[231] Johnson JO, Mandrioli J, Benatar M (2010). Exome sequencing reveals VCP mutations as a cause of familial ALS. *Neuron*.

[232] Fecto F, Yan J, Vemula SP (2011). SQSTM1 mutations in familial and sporadic amyotrophic lateral sclerosis. *Archives of Neurology*.

[233] Roussel BD, Irving JA, Ekeowa UI (2011). Unravelling the twists and turns of the serpinopathies. *The FEBS Journal*.

[234] Kroeger H, Miranda E, MacLeod I (2009). Endoplasmic reticulum-associated degradation (ERAD) and autophagy cooperate to degrade polymerogenic mutant serpins. *Journal of Biological Chemistry*.

[235] Kamimoto T, Shoji S, Hidvegi T (2006). Intracellular inclusions containing mutant *α*1-antitrypsin Z are propagated in the absence of autophagic activity. *Journal of Biological Chemistry*.

[236] Teckman JH, Perlmutter DH (2000). Retention of mutant *α*1-antitrypsin Z in endoplasmic reticulum is associated with an autophagic response. *American Journal of Physiology*.

[237] Narendra DP, Kane LA, Hauser DN, Fearnley IM, Youle RJ (2010). p62/SQSTM1 is required for Parkin-induced mitochondrial clustering but not mitophagy; VDAC1 is dispensable for both. *Autophagy*.

[238] Okatsu K, Saisho K, Shimanuki M (2010). P62/SQSTM1 cooperates with Parkin for perinuclear clustering of depolarized mitochondria. *Genes to Cells*.

